# Cycloalkanes and Aromatics on a Model Astrophysical Surface: Wetting, Dewetting and Thermal Desorption

**DOI:** 10.1021/acsearthspacechem.6c00174

**Published:** 2026-08-21

**Authors:** Rushdi Senevirathne, Martin R. S. McCoustra

**Affiliations:** 3120 Institute of Chemical Sciences, Heriot-Watt University, EDINBURGH EH14 4AS, Scotland

**Keywords:** grain-surface interactions, cycloalkanes, aromatics, wetting and dewetting, temperature-programmed desorption

## Abstract

The interaction of small aromatic molecules (benzene and naphthalene), and of small cycloalkanes (cyclohexane and decalin), with an amorphous silica (aSiO_2_) surface as a model of interstellar silicate grain materials has been explored. Experimental studies of the interaction of cycloalkanes with the aSiO_2_ surface, employing temperature-programmed desorption to explore directly the binding of the adsorbates to the surface, are combined with data derived from previously published studies of the small aromatics. This reveals that, in the monolayer, the aromatic species wet the aSiO_2_ surface while the cycloalkanes tend to dewet. The results of a simple computational comparison of the two reported herein suggest that this behavior results from the donation of charge from the extended π electron structure on the aromatic molecule to Lewis acid Si sites on the aSiO_2_ surface forming a dative interaction and making these Si atoms hypervalent. Such behavior is not possible with the cycloalkanes and their interaction with the aSiO_2_ surface is dominated by weak van der Waals interactions. Reduction of the binding energy of the cycloalkanes compared to the aromatic sees the surface residence time of the former significantly reduced compared to the latter. We speculate as to the possible impact of the role of Lewis acid–base behavior in weakening the bonding in the aromatics consequently reducing the activation energy for their surface hydrogenation in a catalytic fashion.

## Graphical abstract

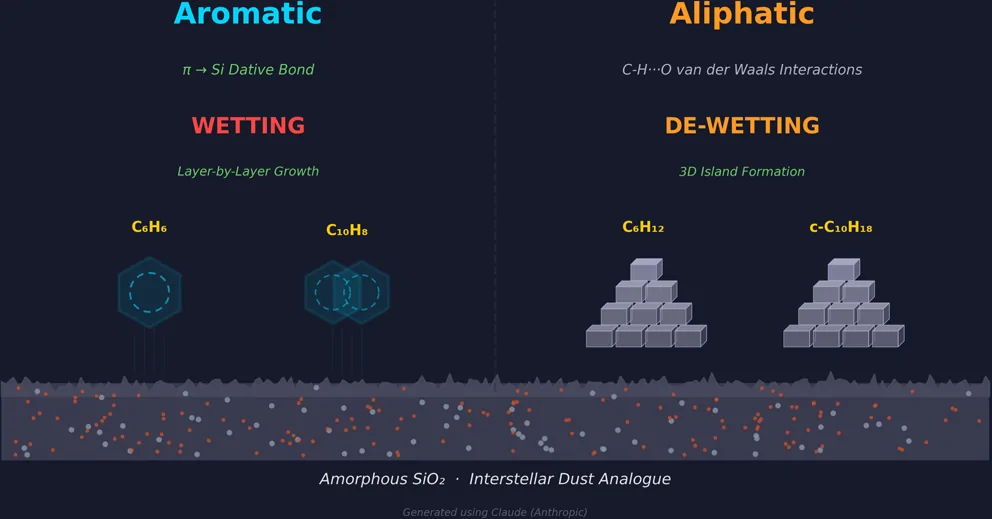

## Introduction

The fundamental mechanisms of thin film growth - Volmer–Weber (island growth in dewetting systems), Frank–van der Merwe (layer-by-layer growth in wetting systems), and Stranski–Krastanov (layer-plus-island growth in systems which change from wetting to dewetting) processes – determine the structure and morphology of thin molecular films on dust grain surfaces in space. These fundamental mechanisms are often defined in terms of wetting versus dewetting processes, and bulk chemical thermodynamics properties such as surface tension and contact angle.^1^ However, they can also be explained in terms of a balance of microscopic forces between adsorbate molecules themselves and between the adsorbate and the substrate. In turn, these growth mechanisms and the resulting film morphologies have an impact on the kinetics of temperature-programmed desorption (TPD) with systems demonstrating Volmer–Weber growth exhibiting zero order desorption at all surface coverages. While Frank-van der Merwe and Stranski-Krastanov grown films will tend to exhibit zero order desorption until the monolayer is accessed when desorption becomes first order.^1^ Both the growth and desorption of thin molecular films on surfaces are underpinned by diffusion. While there are many cutting-edge studies of such in terms of fundamental thin film behavior,^2^ diffusion in astrochemical environments remains relatively poorly investigated as revealed in a recent workshop reported in the literature.^3^

The relevance of these observations to astrochemistry lies in the prevalence of dust in space environments, and its well-recognized roles in promoting the formation of small molecules and the growth of icy films.^4^ This is increasingly important with ongoing discussion on the potentially porous nature of dust in space.^5^ Dust itself is produced in the outflows of old stars and can be a silicate mineral from the olivine and pyroxene families or carbonaceous in nature depending on the type of star.^4^ Growth under the near equilibrium conditions of a stellar atmosphere suggests that these silicate minerals will be crystalline. However, cosmic ray interactions introduce a degree amorphization.^4^ For surface science studies such as in this paper, amorphous silica (aSiO_2_) has proven to be a simple and robust model for silicate minerals in reflecting the Si=O and Si–OH surface chemistry specific to the SiO_4_ framework without the complicating presence of the metal ions. Within this context, the theme of the paper is perhaps best posed in terms of the relative strengths of the interactions of adsorbates with the underlying substrate surface and the intermolecular interactions within the growing icy film. With respect to the silicate mineral surfaces, earlier work has explored the coverage dependent TPD of oxygen (O_2_), nitrogen (N_2_), carbon monoxide (CO) and water (H_2_O) from an amorphous silica (aSiO_2_) film.^6^ In this work, H_2_O desorption was seen to be zero order at all coverages consistent with there being stronger intermolecular interactions in solid water than between water adsorbates and the aSiO_2_ substrate driving dewetting. While for the diatomic species, a transition from first order desorption in the monolayer and below to zero order in the multilayer explores the other extreme where the binding energy of the adsorbate to the aSiO_2_ surface is stronger than the intermolecular interactions in the solid adsorbate.^6^ Consequently, the dewetting behavior of H_2_O on our aSiO_2_ substrate was explained by observations on laboratory time scales of agglomeration of H_2_O into hydrogen-bonded islands at temperatures as low as 18 K.^7^ Carbonaceous grains surfaces, commonly modeled in the laboratory using graphene, graphite and related carbon materials, display consistent zero order desorption kinetics across all coverages for all adsorbates except for aromatic adsorbates. In the latter case, π–π interactions strengthen the interaction with the carbon surface and wetting behavior akin to that discussed for the volatile gases on the aSiO_2_ surface is observed.^8,9^

In contrast, when benzene (C_6_H_6_) adsorption was investigated on our aSiO_2_ substrate the formation of a strongly bound monolayer on the substrate was observed via first order desorption kinetics.^10,11^ Inversion analysis of the C_6_H_6_ TPD data assuming a pre-exponential factor of 1.00 × 10^13^ s^–1^ yields C_6_H_6_ binding energies range from 60 kJ mol^–1^ at low submonolayer coverages to around 43 kJ mol^–1^ as the monolayer is completed. Intermolecular repulsion is known to be responsible for the reduction in binding energy as the surface density of C_6_H_6_ is increased. Given the polar nature of the amorphous silica surface, it is rather chemically counterintuitive to observe dewetting of a polar adsorbate (H_2_O) and wetting by a nonpolar adsorbate (C_6_H_6_). This leads us to the question as to why C_6_H_6_ ^10,11^ (and likely related polycyclic aromatic hydrocarbons (PAHs) such as naphthalene^12,13^ ) should bind so strongly to aSiO_2_?

Benzene is the prototypical (polycyclic) aromatic molecule and has been detected across a wide range of astrophysical environments, including planetary nebula;^14^ post-AGB stars;^15^ the circumstellar envelopes of evolved carbon-rich stars;^16^ comae of comets and asteroids;^17^ the atmospheres of Jupiter, Saturn and its moon Titan;^18^ and most recently, within protoplanetary discs.^19,20^ More recently, two and three ring aromatics have been observed in similar environments.^21–26^ Clearly, these species are readily formed in environments where acetylene is abundant via Diels–Alder chemistry on dust surfaces [e.g., ref 27–29 ]. This represents the foundation for bottom-up growth of polycyclic aromatic hydrocarbons (PAHs), which are a sink for up to a fifth of Galactic carbon.^30^

PAHs are observed in many lines-of-sight as the unidentified IR bands (UIB),^31^ which are more commonly today referred to as the aromatic infrared bands (AIB).^32^ On-going observational, experimental and computational activity continues to strengthen the identification of the AIB carriers as PAHs^33–35^ in much the same way as the PAHs aromatic nature confers sufficient stability to promote their ubiquity.^36,37^ PAHs have been identified as having several important astrophysical and astrochemical roles to play in space. They help identify regions of active star formation by down converting stellar UV into IR.^37–39^ They regulate the energy balance of neutral gas through their photoionization;^40,41^ hence controlling the phase structure of the ISM^42^ as PAH emission depends strongly on the charge on the species.^43^ The charge state itself depends on the rates of photoionization and electron–ion recombination, i.e., the ionization balance; hence PAH IR emission can be used to infer the relevant physical conditions and influence of ambipolar diffusion on the magnetic field that supports dense cores against gravity as well as the ion–molecule chemistry that builds up the small molecules prevalent in these environments.^44,45^ Finally, the laboratory work of Hornekær and co-workers has revealed a crucial role for PAHs in catalyzing the formation of molecular hydrogen (H_2_) via a combination of hydrogenation and superhydrogenation of the PAHs with subsequent loss of hydrogen driven thermally or by radiation for both free flying species and species adsorbed on grains.^46,47^

As C_6_H_6_ is found in dust rich environments, this leads us to speculate as to the impact of superhydrogenation on the interaction of adsorbed C_6_H_6_ (and other PAHs) with an aSiO_2_ substrate as their interaction with carbonaceous materials such as graphene have been reported elsewhere.^48^ In exploring this question, we recognize previous work on wetting and dewetting^6–9^ and report herein (i) an experimental study of the temperature-programmed desorption (TPD) of cyclohexane (C_6_H_12_) and decalin (C_10_H_18_), i.e., superhydrogenated C_6_H_6_ and C_10_H_8_ (naphthalene) from our aSiO_2_ substrate, and (ii) a modest computational exploration of C_6_H_6_ and C_6_H_12_, and C_10_H_8_ and C_10_H_18_, interacting with a simple (SiO_2_)_10_ cluster to reinforce our chemical intuition.

## Methodology

The experiments described herein were conducted in a stainless-steel ultrahigh vacuum (UHV) chamber that has been described elsewhere.^49,50^ The chamber is pumped by a combination of liquid-nitrogen-trapped diffusion pumps and a titanium sublimation pump. Following bakeout to remove water adsorbed on internal surfaces, a 2 × 10^–10^ Torr base pressure is routinely obtainable. The substrate was a 10 mm diameter polished stainless-steel disc attached to molybdenum support posts on an oxygen-free high-conductivity copper mount by tantalum wires. Experiments were conducted on a thin (100–200 nm) film of amorphous SiO_2_ (aSiO_2_) deposited on the polished stainless-steel surface. The aSiO_2_ films were deposited in a separate deposition chamber by electron beam evaporation of bulk fused silica on to a room temperature stainless-steel substrate. The resulting films were characterized morphologically by atomic force microscopy and found to be consistent with the known morphology of interplanetary dust particles.^10,11^ The substrate was maintained at room temperature, and the amount of deposited material was monitored using a quartz crystal microbalance positioned close to the substrate. The film thickness was chosen to ensure a thick enough film to prevent gas-phase access to the stainless-steel substrate without introducing interference effects into infrared measurements. The substrate was cleaned by heating to 500 K for 15 min before cooling, prior to conducting experiments.

The substrate was cooled to a base temperature of around 115 K by a liquid nitrogen reservoir in thermal contact with the sample mount. This temperature is significantly higher than that of dust grains in dense clouds (10–20 K). However, our published work on the interaction of C_6_H_6_ with this type of substrate^10,11^ and preliminary test measurements suggested that C_6_H_12_ does not desorb below 120 K. The substrate was heated resistively by passing current through the support wires. A K-type (Chromel–Alumel) thermocouple, spot-welded to the edge of the substrate, was used for temperature monitoring. A programmable controller (Eurotherm) was used to provide a linear heating ramp (β) of 0.1 ± 0.02 K s^–1^ during temperature-programmed desorption (TPD) experiments.

UV-spectroscopic grade C_6_H_12_ and *trans*-C_10_H_18_ (Sigma-Aldrich) were further purified by repeated freeze–pump–thaw cycles on a dedicated preparation line pumped by a diffusion pump. C_6_H_12_ and *trans*-C_10_H_18_ were deposited on the substrate by backfilling the chamber to a pressure as measured on an uncalibrated ion gauge with the aSiO_2_ substrate at base temperature. Exposures are reported in Langmuir (1 L = 1 × 10^–6^ Torr s). Although it is not possible to accurately determine coverages with our experimental arrangement, the surface concentration of adsorbed molecules was estimated using simple collision theory assuming a unit sticking probability and an ion gauge sensitivity correction factor of 5.4 for C_6_H_12_ and an estimated 12.7 for C_10_H_18_.^51^ The collision frequency, *Z*_*w*_, between gas phase molecules and a surface under a gas pressure, *P*, is given as

1 $$Z_{w}=\frac{P}{\sqrt{2\pi mk_{B}T}}$$

where *m* is the molecular mass of the colliding molecules and *T* is their temperature. The surface density of the adsorbed material, *n*_surf_, is then given by

2 $$n_{surf}=Z_{w}St$$

where *S* is the sticking coefficient and *t* is the dosing time . Table S1 lists the relevant surface concentrations explored in this work calculated using eqs 1 and 2 assuming unit sticking probability. The values have an estimated uncertainty of 20%. The corresponding coverages are, in turn, estimated from the van der Waals areas of the physisorbed molecules on the surface, i.e., 3.90 × 10^–15^ cm^2^ and 5.30 × 10^–15^ cm^2^ respectively for C_6_H_12_ and *trans*-C_10_H_18_.^52^ Table S1 also relates these exposures which range from roughly 0.02 of a close packed monolayer to about 20 layers. For *trans*-C_10_H_18_, the exposures studied only explore coverages in the submonolayer regime. Figure 1 compares the deposited surface concentrations with the integrated TPD yields for each cyclohexane. In being linear, these data indicate that there is no change in sticking coefficient over the entire range of doses employed. A similar trend is observed for *trans*-C_10_H_18_ but is not presented herein.

**Figure 1:**
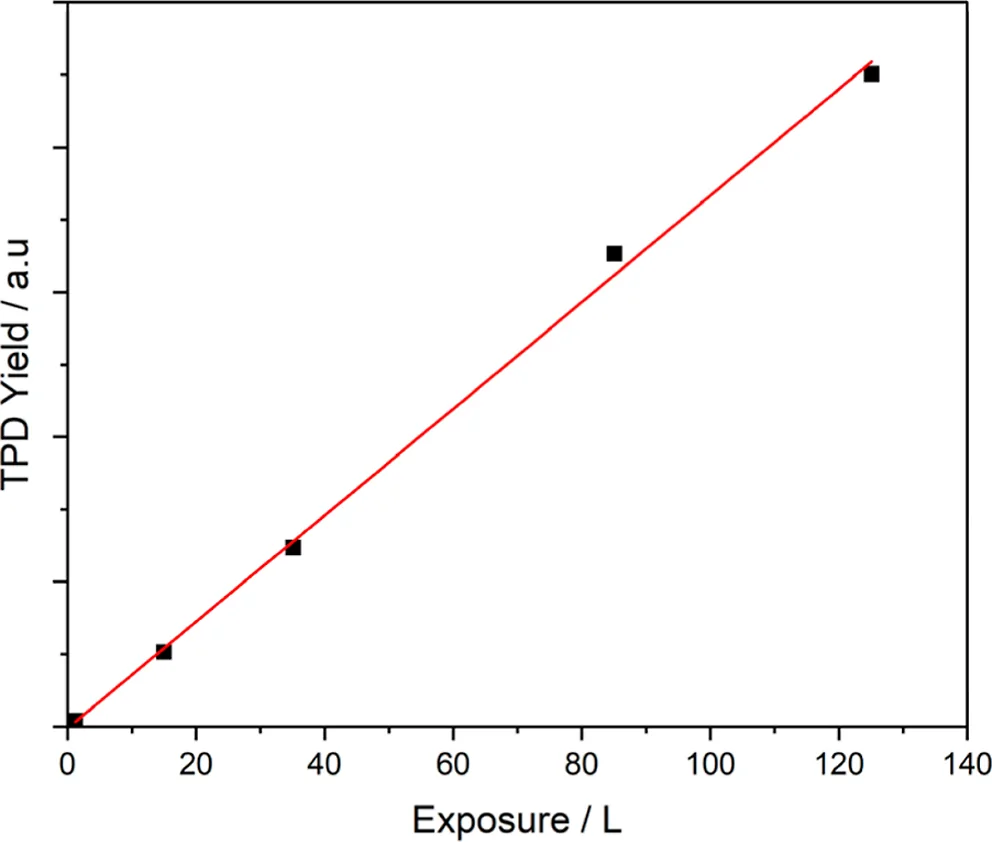
A plot of TPD yield versus exposure for the adsorption of C_6_H_12_ on the aSiO_2_ surface. The red line associated with linear least-squares fitting confirms the linear exposure correlation.

The desorption of C_6_H_12_ and C_10_H_18_ molecules during TPD experiments was monitored using a modified VG Micromass PC300D quadrupole mass spectrometer (QMS) with a crossbeam ion source fitted with a line-of-sight tube and pulse-counting channel electron multiplier detector and using ion counting electronics. The QMS is mounted on a rotary table within the main UHV chamber. This arrangement reduces the background signal from residual gases within the main chamber. During TPD experiments, the substrate was moved close to the opening of this tube to provide a line-of-sight to the QMS ion source. This significantly reduced the detection of molecules desorbing from extraneous surfaces such as the sample mount and support wires and makes the resulting line-of-sight TPD spectra much more open to kinetic analysis.^53,54^ Review of the NIST Chemistry WebBook led us to detect C_6_H_12_ using the ion with an *m*/*z* of 56 m_u_ and C_10_H_18_ with the ion of *m*/*z* of 67 m_u_.^55^ The thermocouple voltage was recorded simultaneously by the QMS software following preamplification and linearization by an integrated circuit and subsequently converted to temperature using the manufacturer’s calibration curve.

To support our experimental work, we have undertaken a modest computational exploration of the cycloalkane versus aromatic interaction with small (SiO_2_)_10_ silica clusters.^56^ Our intention is not to reproduce the experimental work with computations of chemical accuracy. Rather, we chose an approach which would provide us insight into whether there might be a chemical interaction (i.e., electron transfer) between the adsorbates and the substrate and to suggest where that electron transfer might have occurred. Indeed, chemical intuition alone would suggest that electron rich π systems might donate charge to silicon in dative interactions. The low-level calculations we present are simply there to reinforce our chemical intuition. Details of these calculations are given in the Supporting Information

## Results and Discussion

### Qualitative Interpretation of the TPD Data

#### Cyclohexane

TPD of C_6_H_12_ from aSiO_2_ are reported in Figure 2. A range of exposures from 0.15 to 125 L were employed to explore the transition from submonolayer to multilayer regimes. The corresponding coverages reported in Table S1 range from 0.01 ML to just over 8 ML (ML = monolayer).

**Figure 2:**
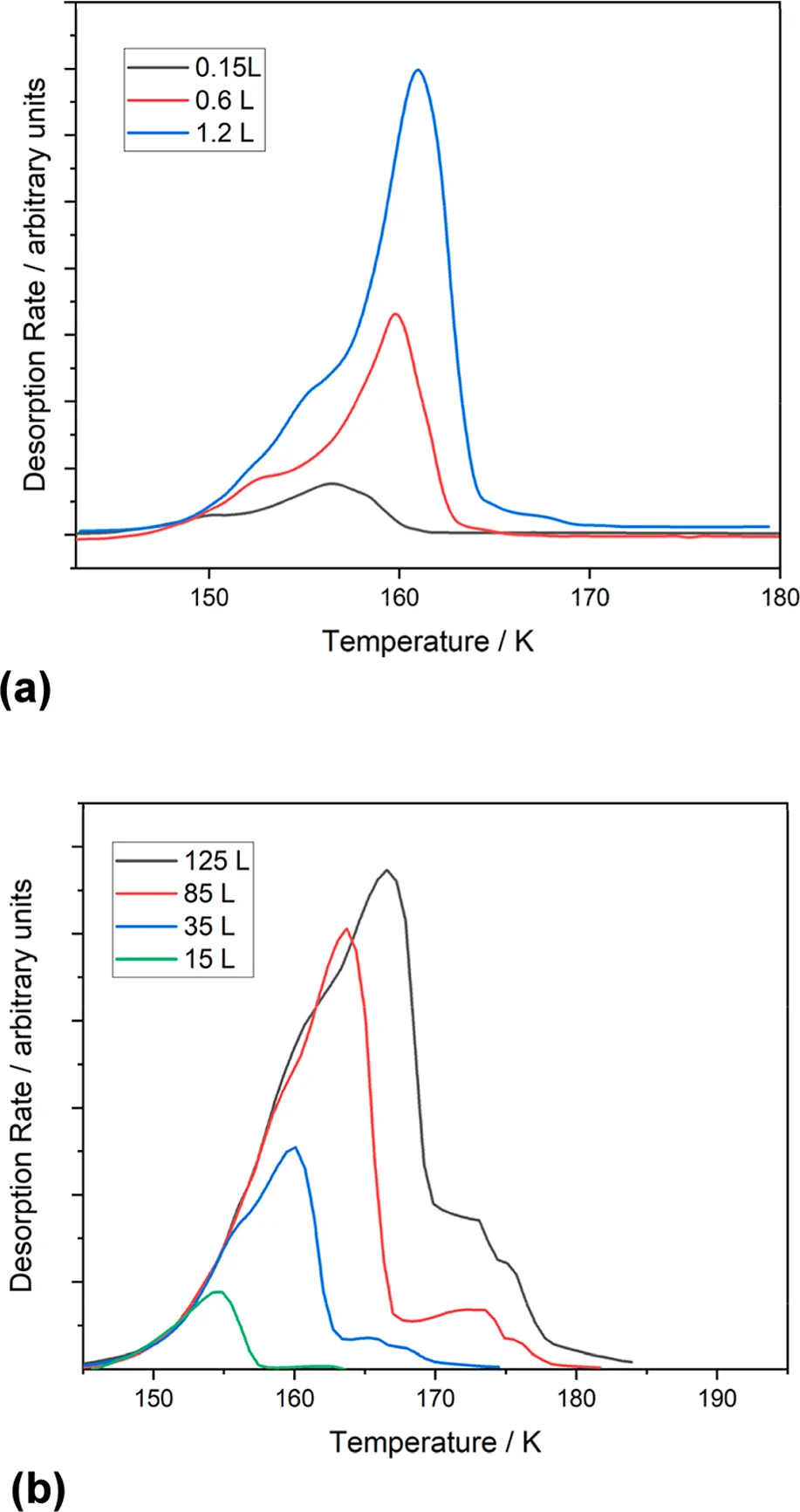
TPD data (averaged) for C_6_H_12_ deposited on an aSiO_2_ substrate, at exposures of (a) 0.15 to 1.20 L, and (b) 15 to 125 L as indicated, at 115 K and heated at 0.1 K s^–1^.

In Figure 2a, corresponding to estimated coverages of 0.02 to 0.20 ML, we observe behavior that is reminiscent of that of the thermal desorption of solid water.^57,58^ The behavior is complex with an initial common leading-edge below 150 K, consistent with zero order desorption, breaking into a shoulder at around 150 to 155 K before a peak in the desorption rate that marches to higher temperature with increasing coverage is observed. The peaked features appear first order but the progression to higher temperature is not consistent with the behavior normally expected for such with increasing surface coverage (i.e., movement to lower temperature). Indeed, peaks moving to higher temperature with increasing coverage are consistent with zero order desorption. This implies Volmer–Weber growth where 3D islands grow on the surface due to rapid diffusive agglomeration while the shoulder is consistent with a phase transition between two solid phases within the growing islands.

As the coverage approaches and exceeds 1 ML, we observe simple zero order behavior with common leading edges (Figure 2b) consistent with a Volmer–Weber type behavior. The high temperature features in Figure 2b support Stranski-Krastanov growth and suggest the presence of some strongly bound C_6_H_12_ at very low coverages on the surface of the aSiO_2_ film hidden under the multilayer film. However, these features, and the curvature away from the exponential leading-edge as the desorption peak is approached, could indicate fractional order desorption from three-dimensional multilayer islands formed by partial dewetting of the multilayer from the aSiO_2_ substrate when adsorbates are mobile on the substrate. This can occur if an equilibrium is established between individual adsorbates and adsorbate islands on a time scale that is fast compared to the desorption rate. As the surface equilibrium must be maintained during the desorption process, the desorption rate will then depend only on temperature and not on coverage. This effect has also been observed for C_6_H_12_ desorption from graphene.^48^

#### Decalin

Decalin (C_10_H_18_) is the two-fused ring analogue of cyclohexane. The bicyclic nature of decalin results in two stereochemical isomers. The positioning of the ring-junction hydrogen atoms determines the stereochemistry and yields *cis*- (where the ring-junction H atoms are the same side of the molecule) or *trans*-decalin (where the ring-junction H atoms are on opposite sides of the molecule). These are noninterconvertible stereoisomers with *trans*-decalin some 3.8 kJ mol^–1^ more stable than the *cis* isomer.^59^

In this work, we have focused on the more stable stereoisomer for consistency with the most stable chair isomer of C_6_H_12_. Figure 3 presents the TPD data for *trans*-C_10_H_18_. It is clear from comparison with Figure 2 that *trans*-C_10_H_18_ behaves differently to C_6_H_12_. At the low exposures, we see a common peak desorption temperature consistent with first order desorption. While at larger exposures, the behavior is more consistent with zero order desorption with a common leading edge and peak temperature moving to higher temperature. This behavior can again be interpreted by considering the potential adjacency of strongly binding sites compared to the size of the adsorbate species. C_6_H_12_ is much smaller than *trans*-C_10_H_18_ and exhibits fewer axial C–H groups on each side of the carbon plane; 3 in chair configured C_6_H_12_ but 5 in *trans*-C_10_H_18_. The latter may permit stronger interaction with the aSiO_2_ surface than the former. Diffusion of the more strongly bound species may be more limited due to larger activation barriers for diffusion because of the larger barrier to desorption. Hence for the extreme low coverage C_10_H_18_ (see Table S1) desorption is faster than diffusion and we observe first order desorption. As the coverage is increased, however, we move to a regime where diffusive agglomeration dominates, as the C_10_H_18_ no longer accesses the strongest bound surface sites, and zero order desorption dominates. With C_6_H_12_, of course, the more limited interaction with the aSiO_2_ surfaces means that we never enter the equivalent phase to the lowest C_10_H_18_ coverages and all we observed is diffusive agglomeration and zero order desorption. This is consistent with Stranski-Krastanov growth, *cf*. C_6_H_12_, especially when the coverages explored are considered and noted to be submonolayer.

**Figure 3:**
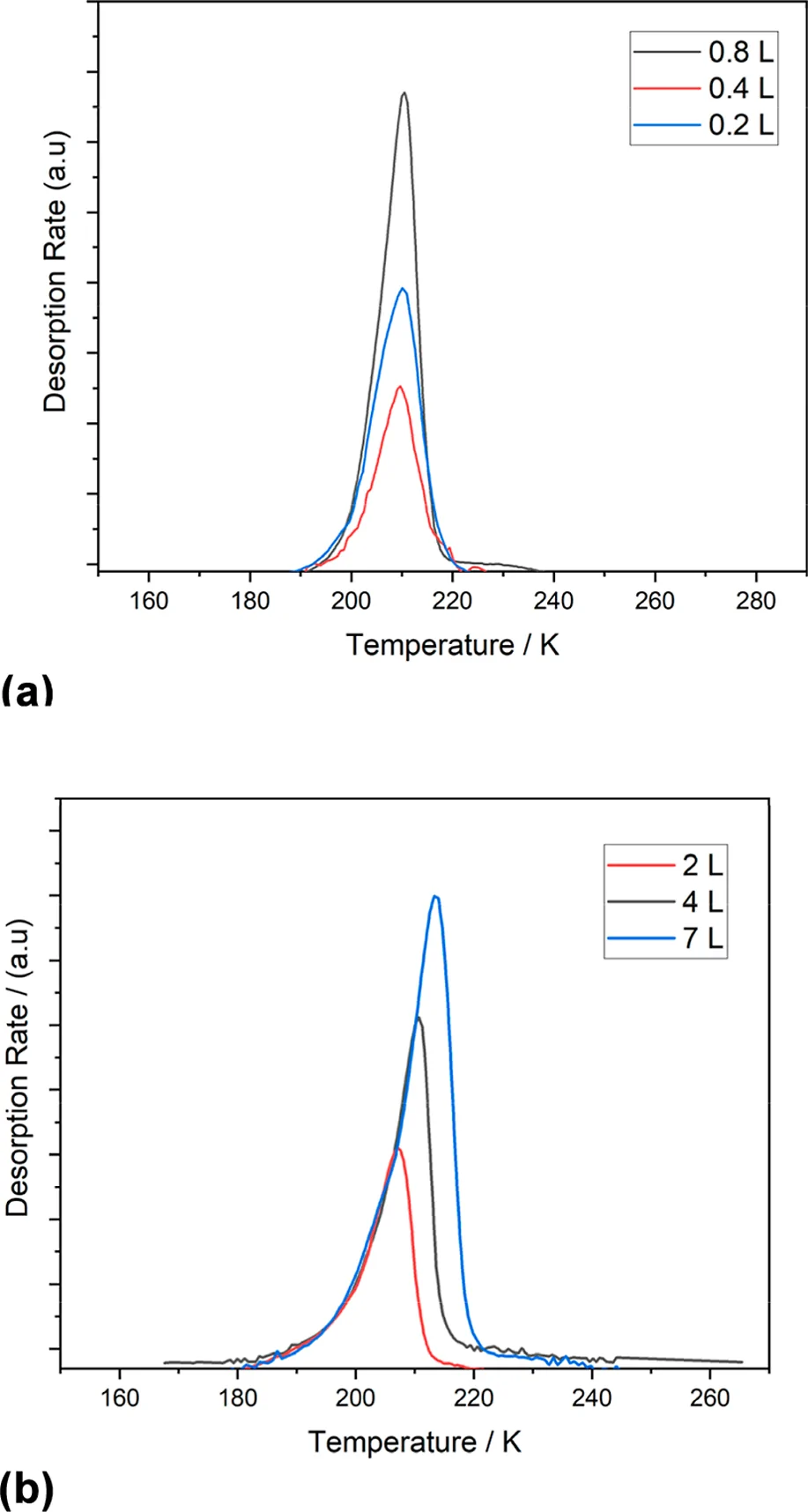
TPD data for *trans*-C_10_H_18_ deposited on an aSiO_2_ substrate, at exposures of (a) 0.2 to 0.8 L, and (b) 2 to 7 L as indicated, at 115 K and heated at 0.1 K s^–1^.

### Quantitative Interpretation of the TPD Data

Interpretation of TPD is founded in the Polanyi–Wigner equation

3 $$-\frac{d\theta }{dt}\left(\theta ,T\right)=r_{des}=\theta^{n}\nu_{des}\left(\theta ,T\right)e^{-E_{des}\left(\theta \right)/k_{B}T}$$

where θ is the coverage, *T* is the temperature, *E*_des_ is the desorption activation energy, *k*_*B*_ is the Boltzmann constant, *ν*_des_ is the pre-exponential factor and *n* is the desorption order (=1 for a first order desorption; 0 for a zero-order desorption).

The first order of the day is confirming the qualitative comments on order made above. This can be achieved through investigations of the initial rate of desorption. By considering TPD data at a fixed temperature for increasing coverages of the adsorption, a log–log plot as represented by eq 4

4 $$\ln\left(r_{des,0}\right)=n\ln\left(\theta_{0}\right)+\ln\left(A_{des,0}\right)-\frac{E_{des}}{RT}$$

of the initial rate of desorption, *r*_des,0_, versus the initial coverage (or exposure), θ_*0*_, yields a straight line whose slope defines the order of desorption. Looking closely at the data in Figure 2a, the desorption rate is essentially the same for all three exposures at 150 K. Logically, therefore, the desorption order is indeed zero. This is equally true for analysis of the data in Figure 2b in the common leading-edge regime. We can conclude therefore that in the low coverage regime we are seeing the effects of a phase transition integrated with zero order desorption *cf*. the situation desorption of amorphous H_2_O.^57,58^

If we are considering zero order desorption, this simplifies the Polanyi–Wigner Equation to

5 $$r_{des}=\nu_{des}\left(\theta ,T\right)e^{-E_{des}\left(\theta \right)/k_{B}T}$$

which implies that a simple Arrhenius Analysis (AA), involving a plot of ln­(*r*_des_) versus 1/*T*, can simultaneously provide *E*_des_ and ν_des_. However, it is bad practice, and prone to substantial error to use the linear extrapolation inherent in AA to estimate ν_des_. Given knowledge of *E*_des_ from AA, ν_des_ can be obtained using a kinetic modeling approach. This allows direct comparison of simulated TPD traces with those obtained experimentally. In this work, kinetic modeling was performed using the Chemical Kinetic Simulator (CKS) software package developed by IBM Almaden Research Centre.^60,61^ The package allows the construction of reaction mechanism based on a series of steps, each with associated Arrhenius parameters, and integrates the associated rate laws using stochastic methods.

To interpret first order desorption data, we have applied Redhead Analysis (RA)^62,63^ as expressed in eq 6

6 $$E_{des}=RT_{p}\left[\ln\left(\frac{\nu_{des}T_{p}}{\beta }\right)-3.64\right]$$

where *T*_*p*_ is the peak desorption temperature, β is the heating rate in K s^–1^ and the pre-exponential factor,ν_des_, was assumed to take its common physisorption value of 1.0 × 10^12^ s^–1^. Once *E*_des_ is defined, we can, as in the case of zero order desorption, undertake kinetic simulations to optimize ν_des_.

#### Cyclohexane

Figure 4 shows the AA for representative exposures of 0.15 and 85 L respectively. The balance of the data can be found in the SI. In developing the analysis of the low exposure data, we have assumed desorption from three-dimensional islands exhibiting a phase transition as per our qualitative interpretation. As such, we would expect two linear sections in the AA. Indeed, this is what we observe in the Figure 4a. As the monolayer is approached and exceeded, simple multilayer desorption is invoked as confirmed by the linear AA plot in Figure 4b for 85 L exposure of the aSiO_2_ surface to C_6_H_12_.

**Figure 4:**
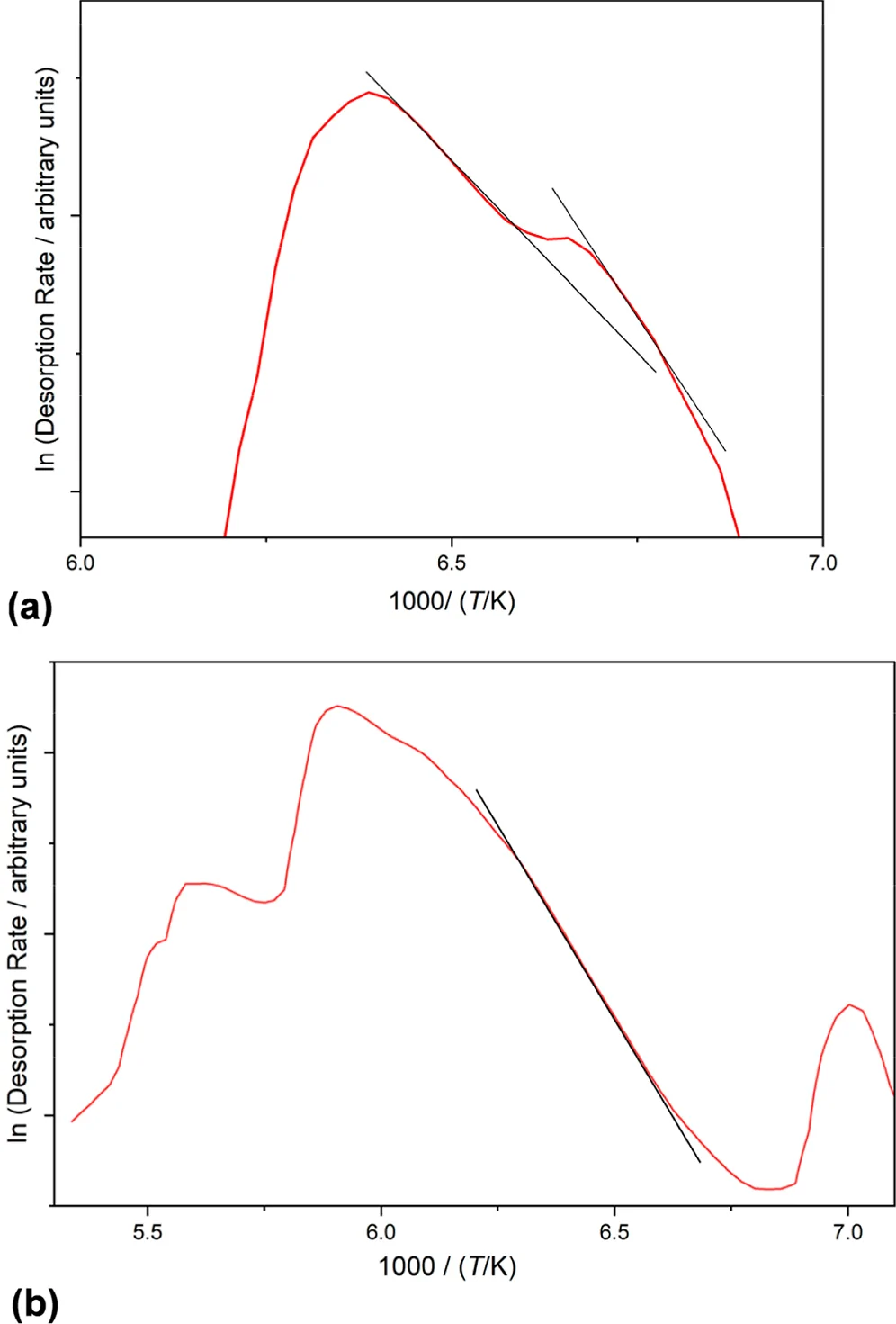
Arrhenius Analysis of the TPD from exposures of (a) 0.15 L and (b) 85 L of C_6_H_12_ on an aSiO_2_ surface. The solid black lines are the result of linear regression fits to the leading-edge data.

Table 1 presents the *E*_des_ values resulting from the AA analysis. The average *E*_des_ for C_6_H_12_ multilayer islands is therefore 44.7 ± 2.8 kJ mol^–1^ between 145 K - 155 K rising to 55.3 ± 1.8 kJ mol^–1^ postphase transition in the temperature range 155 – 165 K. This can be compared to the standard enthalpy of sublimation, Δ_sub_ *H* ^*0*^, reported in the literature of 46.6 kJ mol^–1^ ^64^ at 186 K and a value of 27.6 kJ mol^–1^ at 265 K.^55^ This strong temperature dependence of the quantity is consistent with entropic effects. The difference between the two *E*_des_ averages to 6.7 ± 1.1 kJ mol^–1^ and represents the energy difference between low temperature amorphous phase and the high temperature brittle crystal phase.^64,65^

**Table 1:** Activation Energies for Desorption, *E*_des_, Obtained from AA of the Desorption of C_6_H_12_ from the aSiO_2_ Surface, and Optimized *ν*_des_ for Simulated TPD^a^

C6H12 exposure/L	*E*des/kJ mol^–1^		*ν*des/cm^–2^ s^–1^
0.15	41.5	46.9	1.3 × 10^23^
0.60	45.7	52.9	2.8 × 10^23^
1.2	46.9	54.4	3.3 × 10^24^
15	57.1		2.5 × 10^28^
35	57.3		3.2 × 10^28^
85	56.2		2.4 × 10^28^
125	54.1		4.3 × 10^28^

a The low exposure data are represented by two values consistent with the proposal that there is a phase change occurring in the islanded C_6_H_12_. All exposures to the aSiO_2_ surface were made at base temperature.

Thus, we can interpret the C_6_H_12_ TPD data as reflecting deposition from the gas phase onto a cold substrate at 115 K producing an amorphous solid. At around 150 K, this undergoes an amorphous to brittle crystalline phase transition.

The ν_des_ reported in Table 1 were determined using kinetic analysis assuming a relatively simple reaction mechanism reflecting desorption from multilayer material containing four steps, [Disp-formula uneq1] through [Disp-formula uneq4] as below.

R1 $$C_{6}H_{12}\left(ads-1\right)\to C_{6}H_{12}\left(g\right)$$

R2 $$C_{6}H_{12}\left(ads-2\right)\to C_{6}H_{12}\left(g\right)$$

R3 $$C_{6}H_{12}\left(ads-1\right)\to C_{6}H_{12}\left(ads-2\right)$$

R4 $$C_{6}H_{12}\left(g\right)\to C_{6}H_{12}\left(pumped\right)$$

In this scheme, (ads-1), (ads-2), and (g) indicate the adsorbed states in the low temperature amorphous phase, the high temperature brittle crystal phase, and gas phase C_6_H_12_ respectively, with pumped indicating C_6_H_12_ that has been removed from the UHV chamber by pumping. Each of the steps has an associated rate law which necessarily reflects the zero-order kinetics of [Disp-formula uneq1] and [Disp-formula uneq2], and first order kinetics of the phase transition [Disp-formula uneq3] and removal of gaseous C_6_H_12_ by pumping in R4. Given the similarity in temperature trend of the TPD to that of amorphous water, the kinetics of [Disp-formula uneq3] were taken from our earlier work on water ice as were the pumping kinetics.^66^ The *E*_des_ values were fixed to the values estimated from the AA of the desorption; and the experimental heating curves were used rather than assuming a simple linear heating ramp. Initial C_6_H_12_ surface concentrations were taken from Table S1. The pre-exponential factors for the desorption steps were varied to produce the best fit by eye to the leading-edge shape and peak position for each experimental coverage. The resulting simulations summarized in Figure 5 are in good agreement with experimental observations in Figure 2.

**Figure 5:**
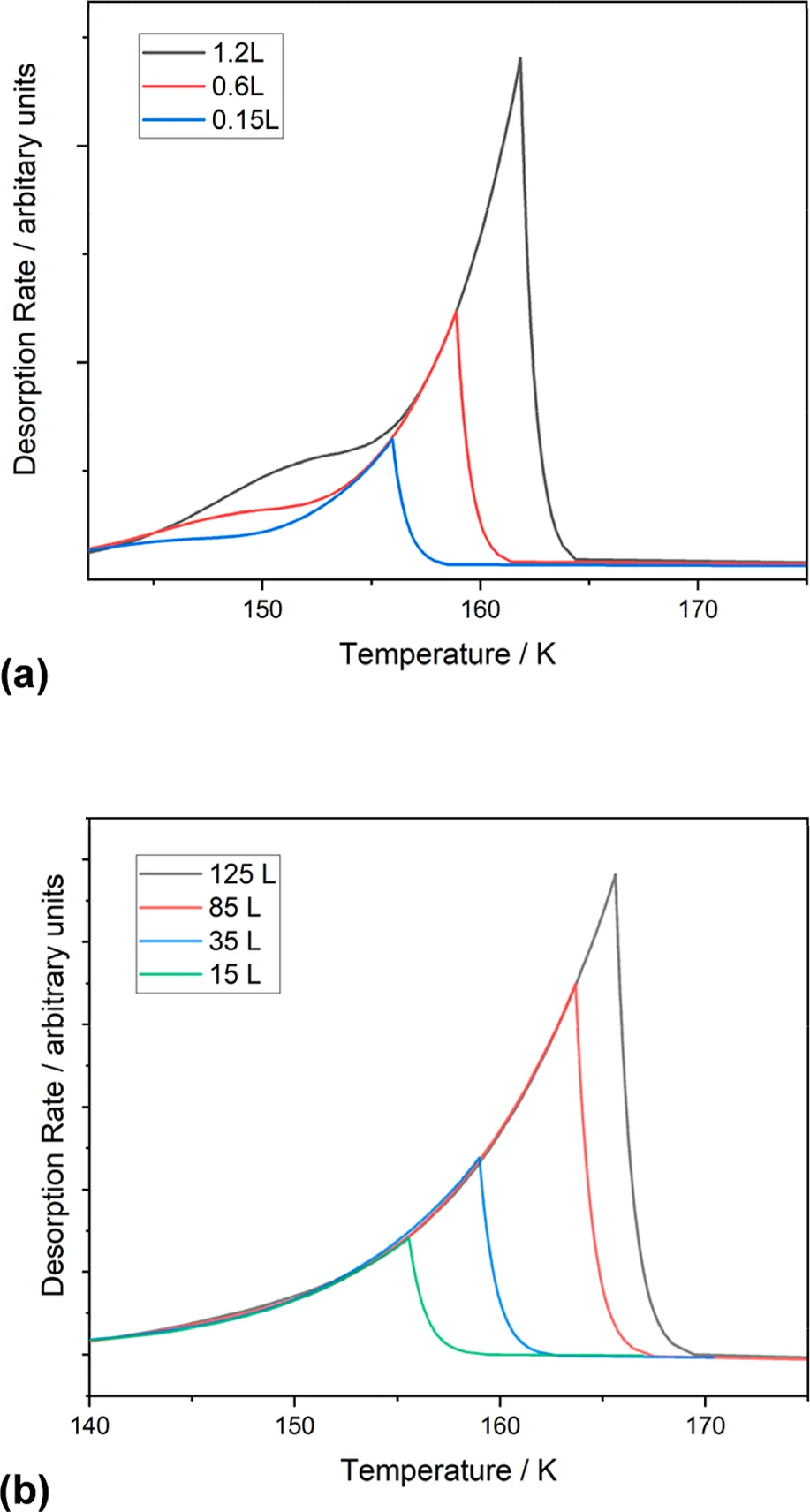
Simulated TPD of (a) 0.15, 0.60, and 1.20 L, and of (b) 15, 35, 85, and 125 L C_6_H_12_ exposures to the aSiO_2_ surface at base temperature.

However, the agreement between the simulations and the experimental trailing edge is less good. But this should come as no surprise as the model has not been constructed to reflect the potentially complex nature of that region of the TPD data as discussed in our qualitative interpretation above. Table 1 also lists the values of ν_des_ recovered from this analysis which yield an average value of (1.2 ± 1.8) × 10^24^ cm^–2^ s^–1^ for the low temperature amorphous phase and (3.1 ± 0.9) × 10^28^ cm^–2^ s^–1^ for the high temperature brittle crystalline phase. Recognizing the uncertainties in the measurements on the desorption of the amorphous phase, we would strongly caution the reader seeking to apply this average value in astrochemical models. This significant difference in ν_des_ between the two phases is not unusual as this quantity is sensitive to the entropy of activation, and as we have already indicated, entropic effects play a significant role in phase transitions and thermal desorption.

Table 2 presents a comparison of the kinetic parameters for multilayer desorption of C_6_H_12_ with those reported in the work of Thrower et al. for C_6_H_6_ on the same substrate.^10,11^ The C_6_H_6_–C_6_H_6_ and C_6_H_12_–C_6_H_12_ interactions probed by multilayer desorption are mainly van der Waals in nature. Though in the case of C_6_H_6_, van der Waals interactions may be augmented by π–π interactions. The observation that the *E*_des_ of C_6_H_6_ is smaller than that of C_6_H_12_ is not inconsistent with the fact that C_6_H_12_ is heavier than C_6_H_6_. The most immediate conclusion that we can draw from this comparison is that the multilayer binding energies of both C_6_H_6_ and C_6_H_12_ are similar within the reported error limits as might be expected given the similarity in mass and the chemical nature of the species. What is starker to compare are the pre-exponential factors, which within a transition state theory model reflect the entropy of activation for desorption. This comparison suggests significant differences in the molecular degrees of freedom of the adsorbed and desorbed states, which contribute toward the entropy change during desorption.

**Table 2:** Comparison of Desorption Rate Parameters for C_6_H_12_ and C_6_H_6_ from aSiO_2_ and Graphene

substrate		*E*_des_/kJ mol^–1^	ν_des_
aSiO_2_	C_6_H_12_ (this work)	55.3 ± 1.8 (multilayer)	3.10 × 10^28^ cm^–2^ s^–1^
	C_6_H_6_ ^10,11^	42.0–60.0 (monolayer)	1.0 × 10^13^ s^–1^
		48.1 ± 2.0 (multilayer)	1.0 × 10^30 ± 0.5^ cm^–2^ s^–1^
Graphene on Pt (111)	C_6_H_12_ ^48^	53.5 ± 2 (multilayer)	1.7 × 10^16 ± 1^ cm^–2^ s^–1^
	C_6_H_6_ ^48^	54.5 ± 2.0 (monolayer)	1.0 × 10^17 ± 1^ s^–1^
		53.0 ± 2.0 (multilayer)	3.20 × 10^18 ± 1^ s^–1^

The impact of data reported above is explored in the Astrophysical Impact and Conclusions below. However, a pertinent comparison to make in the light of the discussion of wetting/dewetting is to compare the C_6_H_6_ *E*_des_ in the monolayer with the data for the C_6_H_12_ multilayer in Table 2 as in relevant astrophysical environments C_6_H_6_ and C_6_H_12_ coverages are likely to be small as there is currently no observational evidence in the infrared of either species in the adsorbed or solid state. Thrower et al. report this quantity as ranging from 60 kJ mol^–1^ at low submonolayer coverage to 42 kJ mol^–1^ at near monolayer coverages reflecting increasing repulsive interactions as the monolayer is approached.^10,11^ This clearly implies that the C_6_H_6_ monolayer is more strongly bound to the substrate than either its own multilayer or the C_6_H_12_ multilayer.

#### Decalin

If we now turn to *trans*-C_10_H_18_, the behavior is somewhat different to that of C_6_H_12_. Significantly, it should be noted that the entire exposure range explored represents submonolayer quantities. However, the data clearly show initial wetting of the aSiO_2_ surface and first order desorption being observed up to coverages of 0.06. Thereafter, island growth takes over and zero order (multilayer) desorption is observed. Table 3 summarizes the results of the analysis of the TPD data for each exposure as detailed below.

**Table 3:** *E*_des_ Values Derived from a Redhead and Arrhenius Analyses and Optimized Pre-exponential Factors Obtained From CKS Analysis for TPD of Various Exposures of *trans*-Decalin Adsorbed on an aSiO_2_ Surface Held at Base Temperature

exposure/L	*E*des/kJ mol^–1^	ν_des_
0.20	65.6 ± 0.3	(3.5 ± 0.2) × 10^12^ s^–1^
0.40	69.5 ± 0.5	(3.0 ± 0.2) × 10^12^ s^–1^
0.80	68.3 ± 0.5	(3.5 ± 0.2) × 10^12^ s^–1^
2.00	64.4 ± 0.3	(2.1 ± 0.2) × 10^26^ cm^–2^ s^–1^
4.00	62.1 ± 0.2	(3.6 ± 0.2) × 10^26^ cm^–2^ s^–1^
7.00	61.2 ± 0.3	(4.8 ± 0.2) × 10^26^ cm^–2^ s^–1^

In the range 0.20 to 0.80 L, RA was carried out for each exposure taking ν_des_ to have the common physisorption value of 1.0 × 10^12^ s^–1^ at each coverage. The resulting average *E*_des_ within this regime is then 67.8 ± 0.4 kJ mol^–1^. CKS analysis then makes it possible to estimate the pre-exponential factor for desorption in this regime. The simple two-step reaction scheme below was utilized and assumed the desorption process is first order rather than zero order. The first order system pumping was retained and initial concentrations obtained from simple collision theory.

R5 $$C_{10}H_{18}\left(ads\right)\to C_{10}H_{18}\left(g\right)$$

R6 $$C_{10}H_{18}\left(g\right)\to C_{10}H_{18}\left(pumped\right)$$

The desorption energy, *E*_des_ was set to 67.8 kJ mol^–1^ as above and the experimental heating curves were used rather than assuming a simple linear heating ramp. The pre-exponential factor for the desorption step was varied to produce the best fit to the peak shape and position at each experimental coverage. Table 3 lists the results of the CKS analysis for each coverage and the corresponding simulations are shown in Figure 6. The resulting average ν_des_ within the submonolayer phase is (3.3 ± 0.7) × 10^12^ s^–1^.

**Figure 6:**
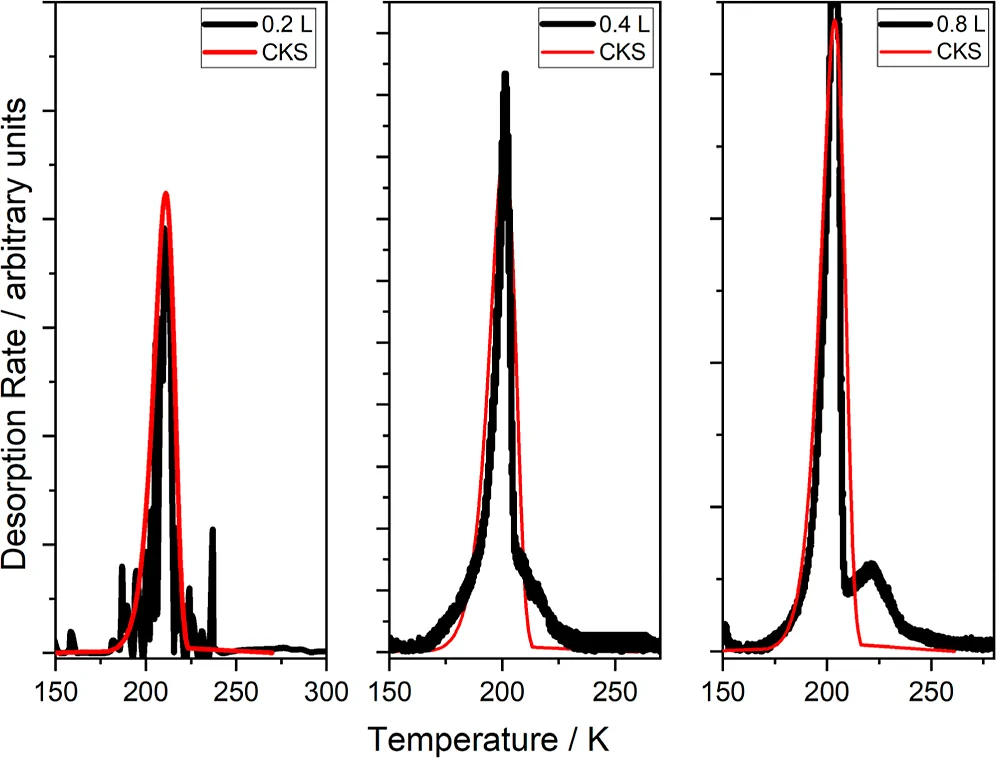
TPD results (experimental data in black) in comparison with the CKS model simulations (red line) for exposures of 0.2, 0.4, and 0.8 L of *trans*-decalin to the aSiO_2_ surface at base temperature.

The results of AA of the TPD for *trans*-C_10_H_18_ within the exposure range 2.00 to 7.00 L which exhibits zero order desorption are also listed in Table 3. The average value of *E*_des_ was found to be 62.6 ± 0.3 kJ mol^–1^. This is not inconsistent with the standard enthalpy of sublimation, Δ_sub_ *H* ^*0*^, reported in the literature of 66.2 kJ mol^–1^.^55,64^ This average *E*_des_ was then used in CKS simulations akin to those for C_6_H_12_ but considering only desorption of a single phase. The experimental heating curves were used rather than assuming a simple linear heating ramp. The pre-exponential factor for the desorption step was varied to produce the best fit to the peak shape and position at each experimental coverage. The results are also summarized in Table 3. The average ν_des_ from the data in Table 3 is (3.5 ± 1.4) × 10^26^ cm^–2^ s^–1^.

Desorption energy values for *trans*-C_10_H_18_ exposures realizing first order desorption kinetics (67.8 kJ mol^–1^) and zero order desorption kinetics (62.6 kJ mol^–1^) occur for coverages substantially below monolayer consistent with an islanded growth mechanism. This reinforces the idea that the interaction of *trans*-C_10_H_18_ with the aSiO_2_ surface is moderated via weak noncovalent (van der Waals) interactions. However, the presence of distinct first layer indicates, and this is confirmed by the data, that the interaction of *trans*-C_10_H_18_ with the aSiO_2_ surface is slightly stronger than within the *trans*-C_10_H_18_ multilayer. *trans*-C_10_H_18_ therefore, at least initially, wets the aSiO_2_ surface before growing as islands.

### A Computational Exploration of C_6_H_*x*_ and C_10_H_*x*_ Binding to (SiO_2_)_10_ Clusters

#### Benzene and Cyclohexane

The most strongly bound C_6_H_6_-(SiO_2_)_10_ and C_6_H_12_-(SiO_2_)_10_ clusters are shown in Figure 7. The corresponding binding energy, *E*_*b*_ as defined by eq S3, calculated after BSSE correction by the CP method using eq S2 are given in the Table 4.

**Figure 7:**
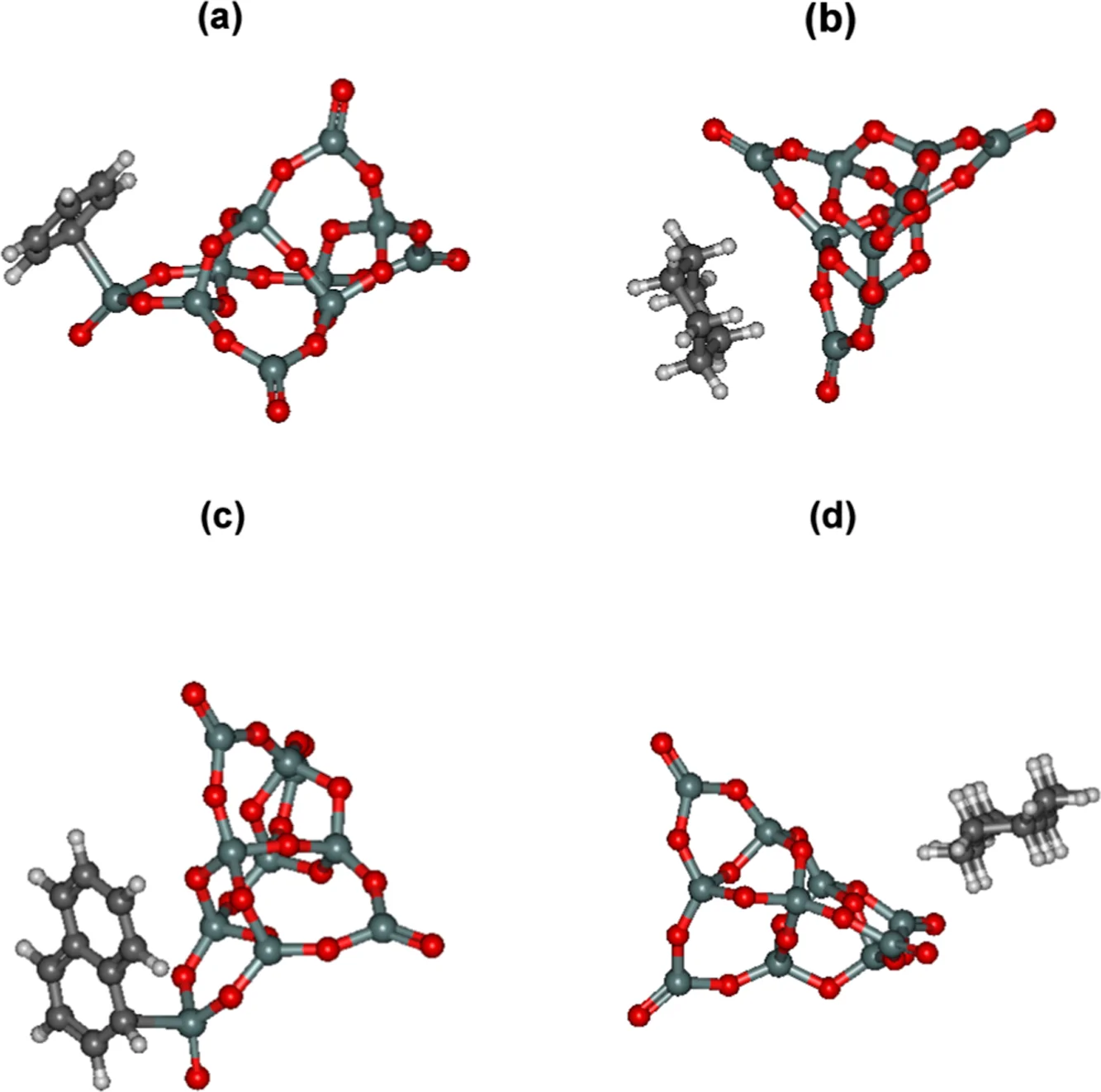
Optimized geometries of the most strongly bound (a) C_6_H_6_-(SiO_2_)_10_, (b) C_6_H_12_-(SiO_2_)_10_, (c) C_10_H_8_-(SiO_2_)_10_, (d) *trans*-C_10_H_18_-(SiO_2_)_10_ clusters. The presence of a bond between the aromatic π system and a Si atom in the cluster surface is evident for both C_6_H_6_ and C_10_H_8_.

**Table 4:** Δ*E*_ads_ and *E*_b_ Calculated using eqs S1 and S3 through^a^

system	Δ*E*_ads_/kJ mol^–1^	*E*_b_/kJ mol^–1^
C_6_H_6_-(SiO_2_)_10_	–76.52	72.22
C_6_H_12_-(SiO_2_)_10_	–11.42	61.36
C_10_H_8_-(SiO_2_)_10_	–124.70	79.28
C_10_H_18_-(SiO_2_)_10_	–2.73	1.32

a All computed energies used in these calculations are corrected for BSSE by the CP method. *E*_b_ includes harmonic zero-point energy corrections, for the most strongly bound C_6_H_6_-(SiO_2_)_10_, C_6_H_12_-(SiO_2_)_10_, C_10_H_8_–(SiO_2_)_10,_ and C_10_H_18_-(SiO_2_)_10_ complexes. Δ*E*_ads_ are referenced to the two components of the cluster held at infinite separation, while the *E*_b_ are reference to the energetic minima.

The first observation we can make on this data is of the strength of the interaction between the C_6_H_6_ moiety and the (SiO_2_)_10_ cluster. At 72.22 kJ mol^–1^, the value reported in Table 4 is not inconsistent with the low coverage limit of *E*_des_ for C_6_H_6_ of 60 kJ mol^–1^ reported in the literature.^8,9^ This may also point to a small or negligible barrier to adsorption for C_6_H_6_ on our aSiO_2_ substrate. Reflecting further on this comparison and the C_6_H_6_–Si bond displayed in Figure 7a; the strength of this interaction arises from partial electron transfer from the C_6_H_6_ π electron density to a sp^2^ hybridized Si atom in an Si=O group on the cluster, i.e., we are seeing Lewis acid behavior on these sites on our aSiO_2_ surface. Such hypervalent behavior is well-known for Si=O moieties and dominates its chemistry.^67–69^ In support of our proposal that there is a dative interaction between the aromatic π electron density and specific sites on our surface, we cite the thesis work of Thrower^70^ wherein the C–H out-of-plane vibration (ν_4_) is found to shift from 689 cm^–1^ in multilayer environments to 680 cm^–1^ in the monolayer regime. This parallels a shift observed in the computed out-of-plane harmonic frequency from 772.68 cm^–1^ in the uncoordinated C_6_H_6_ to 754.45 cm^–1^ with the molecule coordinated to the Si atom in an > Si=O moiety. Such a downward shift in vibrational frequency for the C_6_H_6_ out-of-plane mode is consistent with weakening of the aromatic π system as result of electron donation.

When we consider the C_6_H_12_-(SiO_2_)_10_ cluster, the energy release associated with adsorption, Δ*E*_ads_ from eq S2, yields a value significantly smaller than the corresponding value for C_6_H_6_-(SiO_2_)_10_. However, correction for the zero-point energies of the corresponding component systems results in a stabilization of the cluster and the calculated *E*_*b*_ in Table 4 is not inconsistent with the *E*_des_ determined experimentally. Though, the interaction of the aromatic remains stronger than that of the cycloalkane as consistent with the comparison of wetting versus dewetting behavior in the experimental measurements.

#### Naphthalene and *trans*-Decalin

Turning to the comparison of the interaction of naphthalene (C_10_H_8_) and the quasi-planar *trans*-decalin (C_10_H_18_) isomer with the (SiO_2_)_10_ cluster, the most strongly bound C_10_H_8_-(SiO_2_)_10_ and C_10_H_18_-(SiO_2_)_10_ clusters are shown in Figure 7c,d. The corresponding *E*_*b*_ calculated after BSSE correction by the CP method using eq S3 are given in the Table 4. In this case, Δ*E*_ads_ from eq S2 yields values of −124.70 and −2.73 kJ mol^–1^ for the C_10_H_8_ and C_10_H_18_ clusters, respectively. Interestingly, *E*_*b*_ for the cycloalkane is significantly smaller, at only 1.32 kJ mol^–1^, than the corresponding value for C_10_H_8_ on the (SiO_2_)_10_ cluster (79.28 kJ mol^–1^ as given in Table 4). Unfortunately, we have yet to measure *E*_des_ for C_10_H_8_ on our aSiO_2_ surface and must turn to the literature for comparative values. Here we find *E*_des_ ranging from 50 to 100 kJ mol^–1^ for a variety of silica surfaces.^12,13,71,72^ This is not inconsistent with our computed value of *E*_*b*_.

However, these data cannot explain the experimental observation of wetting behavior in *trans*-C_10_H_18_ monolayers. We speculate that cluster size employed in these calculations may be insufficient to accurately represent the interactions between the quasi-planar cycloalkane and the surface of an extended aSiO_2_ film.

## Astrophysical Impact and Conclusions

Table 5 summarizes the results obtained from the analysis of the TPD measurements reported previously and presented herein. The work reported herein helps us reconcile the experimental observations on wetting versus dewetting in the C_6_H_6_/C_6_H_12_ and C_10_H_8_/*trans*-C_10_H_18_ systems, and likely in heavier PAHs. The strong bonding interaction between the electron rich aromatics (C_6_H_6_ and C_10_H_8_) and the Si=O moiety on the (SiO_2_)_10_ cluster supports the formation of an aromatic monolayer and wetting of our aSiO_2_ substrate. The key question then becomes whether such Si=O moieties might be found on silica and silicate mineral surfaces more widely. Si=O moieties have indeed been observed in mechanically activated silica^73^ and their formation has been explained through strain in the surface induced by that mechanical action.^74^ Furthermore, these moieties have received much attention in the computational materials science literature as one of the many functionalizations of silica and silicate surfaces.^75,76^ The presence of Si=O moieties on dust surfaces is crucial in terms of understanding the interaction of PAHs with silicate surfaces as these moieties are clearly open to dative coordination through Lewis Acid–Base driven hypervalent behavior. Their presence will not only enable the coordination of PAHs but will likely extend such coordination to other electron rich π systems.

**Table 5:** Desorption Parameters for the Aromatic (C_6_H_6_ and C_10_H_8_) and Cycloalkane (C_6_H_12_ and C_10_H_18_) Species Discussed in this Work^a^

adsorbate	desorption order	E_des_ from a SiO_2_/kJ mol^–1^	ν_des_ from a SiO_2_
C_6_H_6_	1 ^10,11^	42.0–60.0	1.0 × 10^13^ s^–1^
	0 ^10,11^	48.1 ± 2.0	1.0 × 10^30 ± 0.5^ cm^–2^ s^–1^
C_6_H_12_	0 [this work]	55.3 ± 1.8	3.10 × 10^28^ cm^–2^ s^–1^
C_10_H_8_	1 ^12,13,71,72^	<100	Not Available
C_10_H_18_	1	67.8 ± 0.4	(3.3 ± 0.7) × 10^12^ s^–1^
	0	62.6 ± 0.3	(3.5 ± 1.4) × 10^26^ cm^–2^ s^–1^

a These are the desorption order, *E*_des_ values and optimized pre-exponential factors adsorbed on an aSiO_2_ surface held at base temperature.

In contrast to the aromatics, our study would suggest that the superhydrogenation of a simple aromatic (C_6_H_6_ and C_10_H_8_) will result in the loss of dative electron donation of π electron density to Si in Si=O structures. This reduces the interaction of the corresponding cycloalkane (C_6_H_12_ and *trans*-C_10_H_18_) to simple noncovalent interactions consistent with the relatively easy dewetting from that substrate that we observe experimentally in this work. Both our experimental observations and our computational study support this proposition. This perhaps points to a generalization of applicability of our observations across the PAH family, i.e., PAH wetting on silicaceous surfaces and per-hydrogenated-PAH dewetting. The latter potentially leading to agglomeration of these materials on grain surfaces, as in the case of water^7^ on this substrate, and the presence of adsorbate-free grain surface.

Although the decrease in *E*_des_ from the aromatic to the cycloalkane is relatively small, it has a substantial impact on the residence time, τ_surf_, of the species on the silica surface as illustrated in the following. Surface residence times are defined in terms of the rate constant for desorption, *k*_des_. For a first order desorption, we can define, τ_surf_, as

7 $$\tau_{surf}=\frac{1}{k_{des}}$$

where *k*_des_ is

8 $$k_{des}=\nu_{des}e^{-E_{des}/RT}$$

which is applicable to our aromatic wetting adsorbates C_6_H_6_ and C_10_H_8_. However, for a zero-order desorption process as followed by our dewetting cycloalkane adsorbates, *k*_des_ becomes dependent on the surface density of the adsorbate, *n*_surf_

9 $$k_{des}=\nu_{des}n_{surf}e^{-E_{des}/RT}$$

such that τ_surf_ is

10 $$\tau_{surf}=\frac{1}{k_{des}n_{surf}}$$

This is illustrated in Figure 8 where we have computed the surface residence time for C_6_H_6_ and C_6_H_12_ as a function of surface temperature based on the kinetic data (*E*_des_ and ν_des_) reported herein and considering a variety of surface coverages.

**Figure 8:**
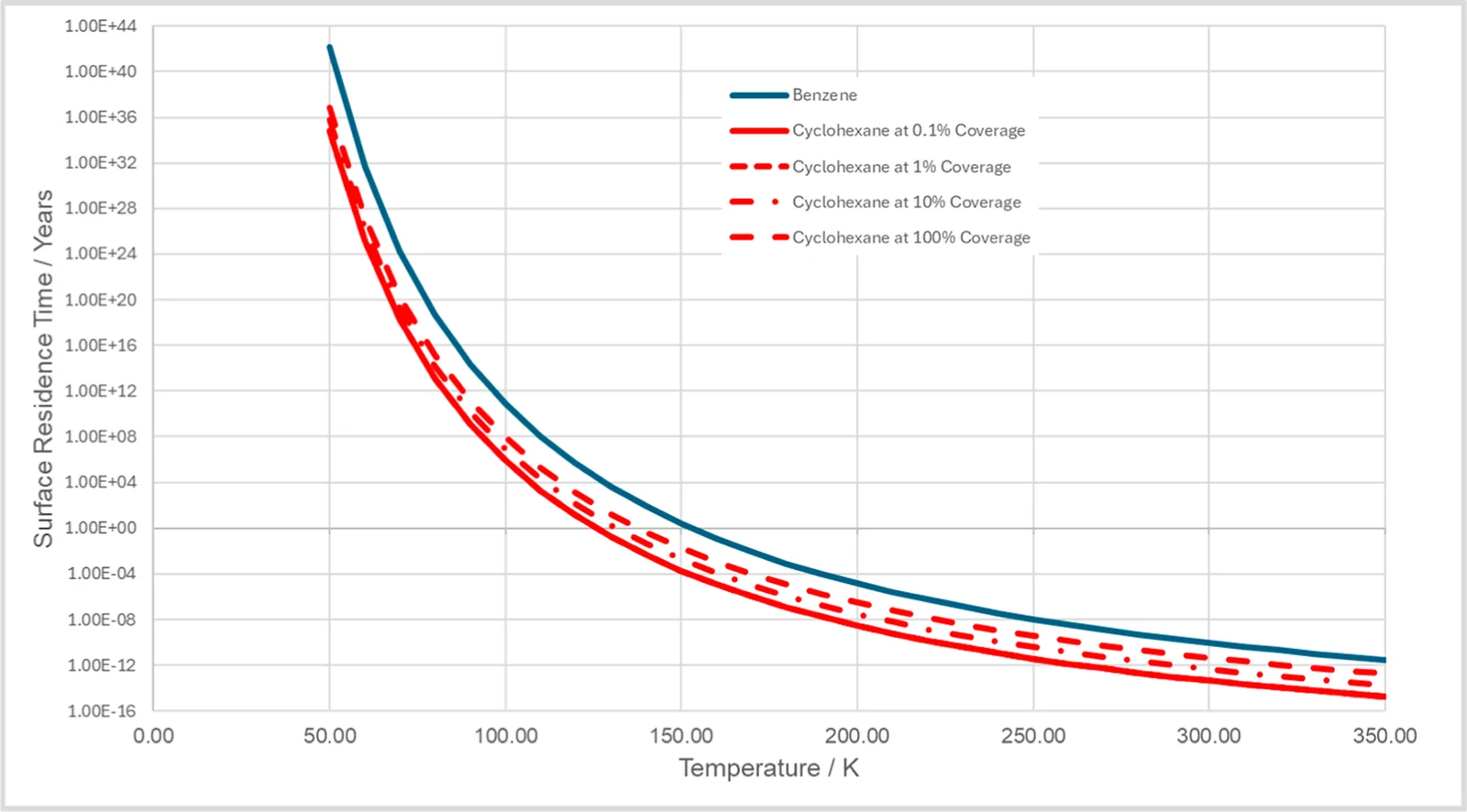
Surface residence time on a SiO_2_ for C_6_H_6_ and C_6_H_12_ computed as a function of surface temperature assuming the kinetic data in Tables 2 and 5

The small difference in the *E*_des_ results in 4 orders of magnitude reduction in residence time at any surface temperature in moving from C_6_H_6_ to C_6_H_12_. Of course, under typical cold, dense environment conditions (i.e., temperatures below 50 K) the residence times are so long as that this difference is pretty much irrelevant as both adsorbates have multimillennial surface residence times. However, if we consider a warm, dense environment as might be found in a protoplanetary disk, this difference becomes significant as it might permit an adsorbed aromatic species to remain on the surface long enough to be fully hydrogenated. While the fully hydrogenated species likely flies off the surface almost as soon as it is formed. Of course, what happens to species with intermediate degrees of hydrogenation (cyclohexadiene and cyclohexene in the case of C_6_H_6_) is worth considering. Such species possess electron rich π systems and it is very likely π donation and hypervalent interactions observed between the aromatics and Si in Si = O moieties will persist into the partially hydrogenated species ensuring their retention on the surface until fully hydrogenated.

It is interesting to speculate on the impact of the donation of π electron density to Si in Si = O moieties on the aromatic species. By breaking the aromatic π ring, could this potentially reduce the activation barrier to hydrogenation of the aromatic consequently increasing the rate of hydrogenation? Are these Lewis acid sites on the aSiO_2_ surface then capable of acting as a hydrogenation catalyst for π bond containing species? If they are, the observations in this paper perhaps suggest a coupled surface-gas phase chemistry, as proposed in Figure 9, linking grain surface hydrogenation of the aromatics (illustrated by the case in C_6_H_6_ in the figure) to rapidly desorbing per-hydrogenated species undergoing gas phase dehydrogenation returning the carbon ring to its aromatic form. The net effect of these coupled cycles would be to provide an additional, high temperature route, converting H atoms to gaseous H_2_. This proposal clearly demands further exploration.

**Figure 9:**
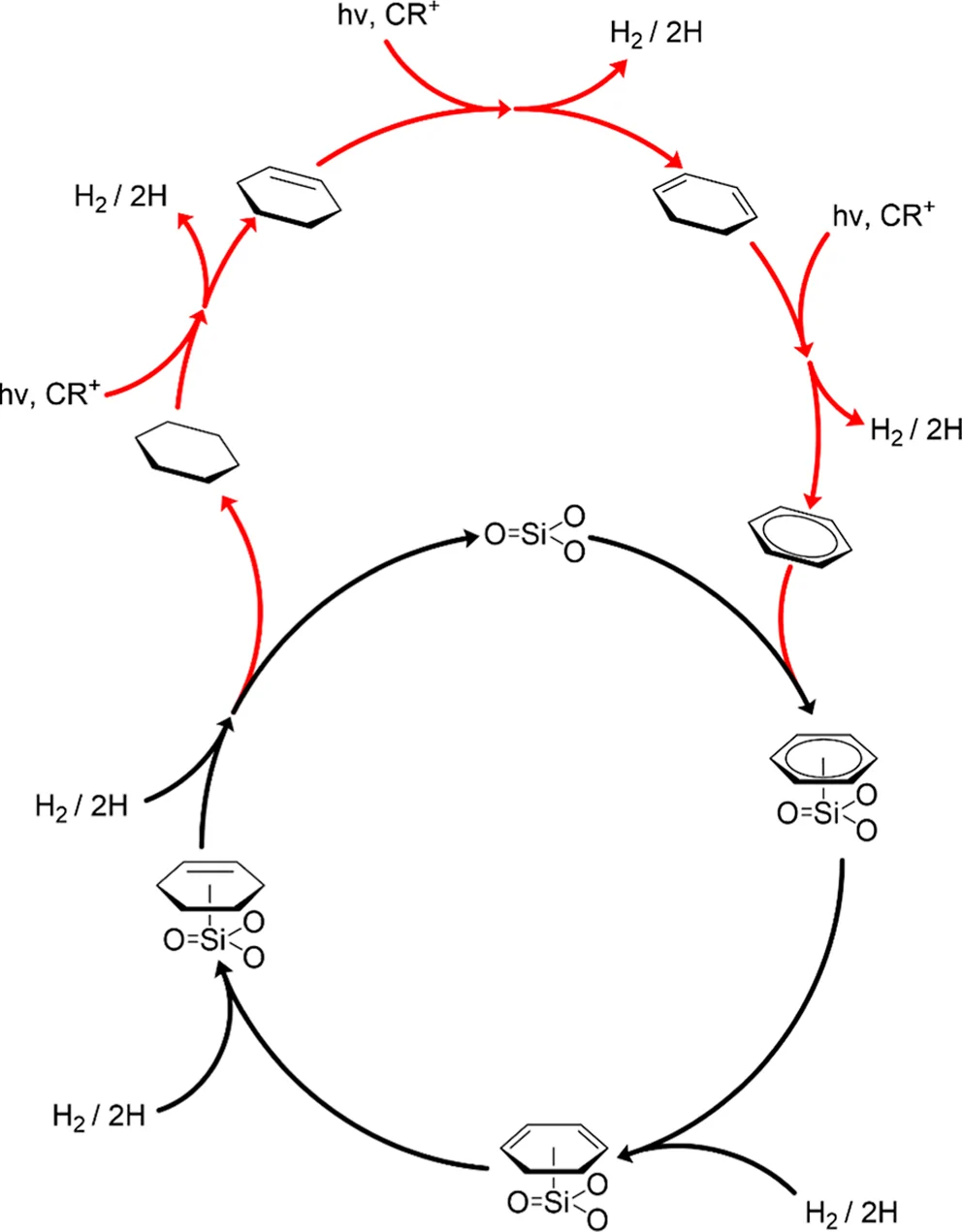
Coupled reaction cycles joining surface-catalyzed hydrogenation of aromatics with gas-phase photolytic or radiolytic dehydrogenation of cycloalkanes.

## Supplementary Material



## Notes

Details of adsorbate exposures (Table S1); Structure of the cycloalkanes and aromatics investigated in this work (Figure S1). Details of computational approach including all optimized geometric information for the isolated cycloalkanes (cyclohexane, C_6_H_12_, and *trans*-decalin (C_10_H_18_) and aromatics (benzene, C_6_H_6_, and naphthalene, C_10_H_8_), the (SiO_2_)_10_ cluster, and the optimized weakly bound complexes derived from these species (Tables S2 through S9). Table of total energies and zero-point energies for all species explored computationally (Table S10). Complete TPD Arrhenius analysis for C_6_H_12_ (Figure S2) and C_10_H_18_ (Figure S3). Optimized kinetic simulations for TPD of C_10_H_18_ multilayers (Figure S4) (PDF)
