# Supplementary material for: Cycloalkanes and Aromatics on a Model Astrophysical Surface: Wetting, Dewetting and Thermal Desorption
Source: ACS Earth Space Chem. 2026 Aug 21;10(9):2072–84. doi: 10.1021/acsearthspacechem.6c00174 (PMC13592367; doi:10.1021/acsearthspacechem.6c00174)
Supplement: Supplementary file 1 [file sp6c00174_si_001.pdf]

Supplementary Information

To

Cycloalkanes and Aromatics on a Model Astrophysical Surface: Wetting, De-wetting and Thermal Desorption

Rushdi Senevirathne and Martin R. S. McCoustra*

Institute of Chemical Sciences, Heriot-Watt University, EDINBURGH, EH14 4AS, Scotland

This document contains supplementary information relevant to the main text of the main paper. The section headings follow those of the main paper.

Methodology

Experimental

Table S1 lists the equivalent surface concentrations of the adsorbates used in this work at the exposures lists in the main text.

Table S1: Initial surface concentrations of C_6H_{12} and of the $C_{10}H_{18}$ isomers employed in this work estimated from the collision frequency on the surface. I monolayer is assumed to represent a close packed layer of C_6H_{12} with a surface density of 3.90×10^{15} cm^{-2} and a close packed layer of $C_{10}H_{18}$ with a surface density of 5.30×10^{15} cm^{-2}. The uncertainty in surface coverage we estimate to be around 20%.

**Exposure / L**	**Surface Concentration / molecules cm^{-2}**	**Surface Coverage / ML**
C_6H_{12}		
0.15	6.25×10^{12}	2.44×10^{-2}
0.60	2.50×10^{13}	9.74×10^{-2}
1.2	5.00×10^{13}	1.95×10^{-1}
15	6.25×10^{14}	2.44
35	1.46×10^{15}	5.68
85	3.54×10^{15}	13.80
125	5.20×10^{15}	20.30
trans-$C_{10}H_{18}$		
0.20	2.97×10^{12}	1.58×10^{-2}
0.40	5.95×10^{12}	3.15×10^{-2}
0.80	1.19×10^{13}	6.30×10^{-2}
2.0	2.97×10^{13}	1.58×10^{-1}
4.0	5.95×10^{13}	3.15×10^{-1}
7.0	1.04×10^{14}	5.52×10^{-1}

Computational

Optimised structures, derived from several starting geometries, and cluster binding energies were calculated for binary clusters of C_6H_x (x = 6, 12) with small $(SiO_2)_{10}$ silica clusters derived from the work of Bromley and co-workers [1] using Hartree-Fock methods with the GAMESS-US 2019 R1 code [2, 3] and GabEdit visualisation tool [4] operating on Windows 10. Calculations were performed on a HP EliteDesk 800 G5 Small Form Factor PC with a 6 core Intel i7-8700 processor operating at 3.2GHz with 32 GB RAM and a 1TB SSD at the RHF level using the 6.311 G** basis set as a demonstration that even moderate compute power can deliver useful results to aid in the interpretation of experimental observations.

Binding energies, E_b, were calculated from the total energies of the cluster, $E_{cluster}$, and of the individual cluster components, *i.e.*, of the hydrocarbon, E_{HC}, and of the silica cluster, E_{SC}, according to the relationship,

$$\Delta E_{ads} = E_{cluster} - (E_{HC} + E_{SC})\,. \tag{S1}$$

In calculating the total energies, basis set superposition error (BSSE) [5, 6] was accounted for using the Counterpoise Method (CP) [7]. The resulting energies were then corrected for harmonic zero-point energies according to **Equation S2**.

$$\Delta U_{ads} = \Delta E_{ads} + \Delta ZPE\,. \tag{S2}$$

Hence,

$$E_b = -\Delta U_{ads}\,. \tag{S3}$$

In the case where there is no activation barrier to adsorption, as is typical of the physisorption process explored in this work,

$$E_{des} = -E_b\,. \tag{S4}$$

Figure S1 below shows the molecular structures for cyclohexane (C_6H_{12}) and *trans*-decalin ($C_{10}H_{18}$) discussed in the main text.

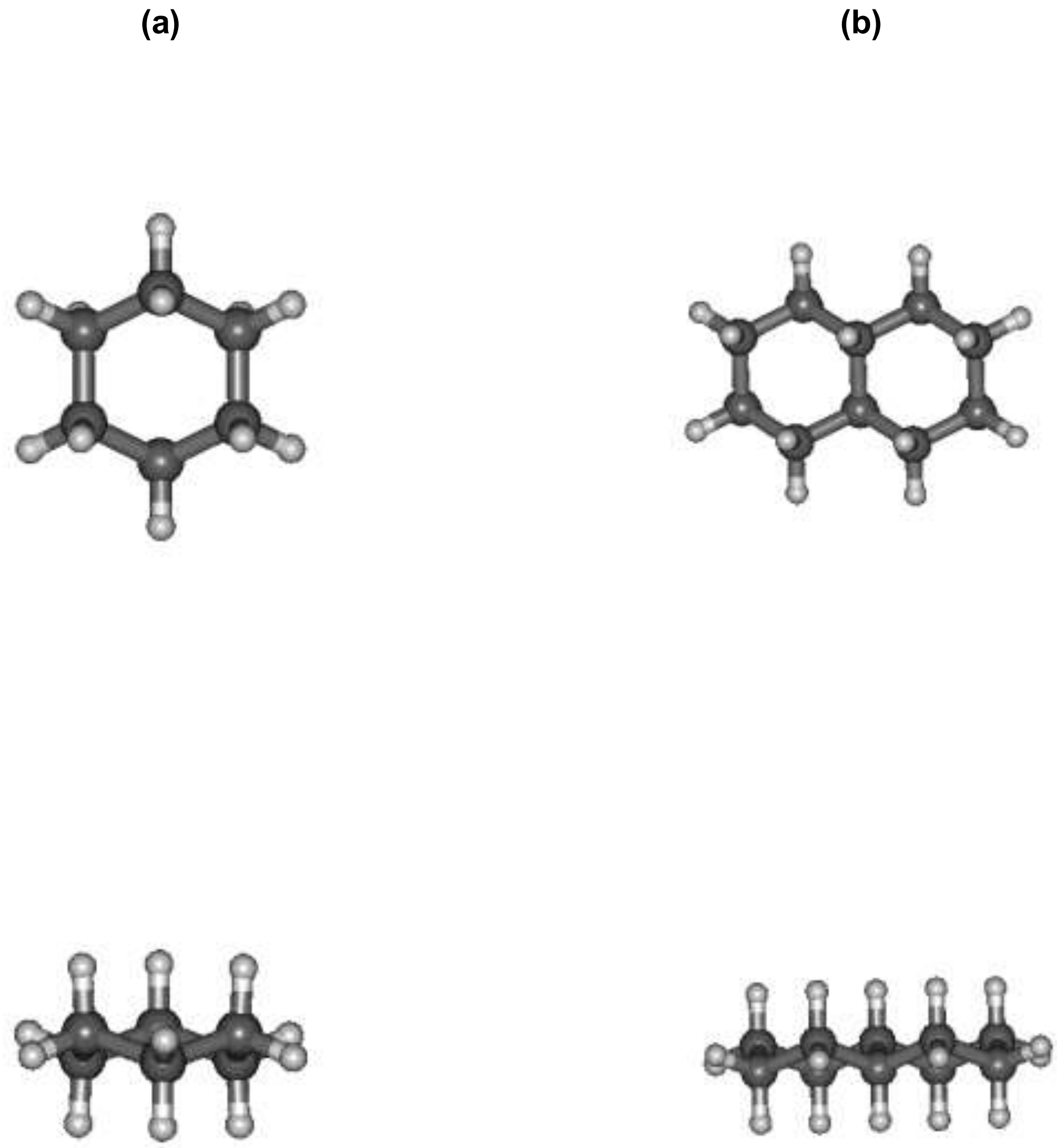

Figure S1: Molecular geometries of **(a)** cyclohexane (C_6H_{12}) and **(b)** *trans*-decalin ($C_{10}H_{18}$) viewed from the top and side to illustrate potential non-covalent interactions.

Optimised Geometries

Tables S2 through **S6** present the optimised geometries of the various monomers considered in the paper.

Table S2: Atom types, atomic numbers, and coordinates in atomic units of the optimised structure of benzene (C_6H_6) obtained from the calculations described in the main text.

Atom	Z	Coordinates / Bohr		
		X	Y	Z
C	6	1.5037286677	0.5137749221	15.0376740713
C	6	1.4336821946	2.9811372319	15.9235624965
C	6	2.0619480553	3.4974821814	18.4168083832
C	6	2.7602547201	1.5464799388	20.0241469474
C	6	2.8302974139	-0.9208785916	19.1382509633

C	6	2.2020258839	-1.4372311000	16.6450088560
H	1	1.0188741122	0.1153205284	13.1137308151
H	1	0.8947701374	4.4866857058	14.6832389547
H	1	2.0078546489	5.4014548133	19.1004413257
H	1	3.2450393558	1.9449570092	21.9481185495
H	1	3.3691187642	-2.4264176169	20.3786009612
H	1	2.2560323629	-3.3412301881	15.9613778032

Table S3: Atom types, atomic numbers, and coordinates in atomic units of the optimised structure of cyclohexane (C_6H_{12}) obtained from the calculations described in the main text.

Atom	Z	Coordinates / Bohr		
		X	Y	Z
C	6	-0.4306345375	-1.5090879306	-2.3194836924
C	6	0.4321274211	1.2543018449	-2.4662417023
C	6	-0.4328001635	2.7628644317	-0.1471132784
C	6	0.4305778458	1.5089499806	2.3195611712
C	6	-0.4321519875	-1.2544492435	2.4663191811
C	6	0.4327718176	-2.7630023817	0.1471831983
H	1	0.2832661461	-2.5555180272	-3.9282074257
H	1	-2.4791768767	-1.5829478709	-2.4325233215
H	1	-0.2807017879	2.1240898047	-4.1773829150
H	1	2.4807453493	1.3158256539	-2.5851734971
H	1	0.2795887393	4.6798421579	-0.2488844715
H	1	-2.4814502171	2.8960598782	-0.1548517063
H	1	-0.2832926023	2.5553838566	3.9282924634
H	1	2.4791239644	1.5828118106	2.4326083592
H	1	0.2806602140	-2.1242372033	4.1774641733
H	1	-2.4807718054	-1.3159617142	2.5852547553
H	1	-0.2796265339	-4.6799763285	0.2489543913
H	1	2.4814199815	-2.8961978282	0.1549329646

Table S4: Atom types, atomic number, and coordinates in atomic units of the optimised structure of naphthalene ($C_{10}H_8$) obtained from the calculations described in the main text.

Atom	Z	Coordinates / Bohr		
		X	Y	Z
C	6	-3.6219811089	5.5873528279	0.0000000000
C	6	-6.1422519669	4.1322260228	0.0000000000
C	6	-6.1422519669	1.2219913100	0.0000000000
C	6	-3.6219811089	-0.2331354951	0.0000000000
C	6	-1.1017102508	1.2219913100	0.0000000000
C	6	-1.1017102508	4.1322260228	0.0000000000
C	6	1.4185606072	5.5873528279	0.0000000000
C	6	1.4185606072	-0.2331354951	0.0000000000
C	6	3.9388314652	1.2219913100	0.0000000000
C	6	3.9388314652	4.1322260228	0.0000000000
H	1	-3.6156108426	-2.2538459499	0.0000000000
H	1	-7.8885420830	0.2057760423	0.0000000000
H	1	-7.8885420830	5.1484412905	0.0000000000
H	1	-3.6156108426	7.6080632827	0.0000000000
H	1	5.6851215813	5.1484412905	0.0000000000
H	1	5.6851215813	0.2057760423	0.0000000000
H	1	1.4121903409	-2.2538459499	0.0000000000
H	1	1.4121903409	7.6080632827	0.0000000000

Table S5: Atom types, atomic number, and coordinates in atomic units of the optimised structure of trans-decalin ($C_{10}H_{18}$) obtained from the calculations described in the main text.

Atom	Z	Coordinates / Bohr		
		X	Y	Z
C	6	1.7765729750	2.2642224353	0.1626959589
C	6	-0.7211515623	3.2968008404	-0.8775377261
C	6	-0.4276638883	4.2184957936	-3.6073017174
C	6	0.6878319136	2.1541836911	-5.3047159605
C	6	3.1967360699	1.1308913995	-4.2705577446
C	6	2.8857835483	0.1864328070	-1.5295328761
C	6	5.3946877045	-0.8368594845	-0.4953746601
C	6	4.3059447534	-0.9469001185	-5.9627865796
C	6	6.8036711804	-1.9794747441	-4.9225528946
C	6	6.5101816167	-2.9011734768	-2.1927907930
H	1	3.1342088164	3.7979712901	0.3164308372
H	1	1.4994597767	1.5357821999	2.0580041972
H	1	-1.4030402877	4.8183987571	0.3114495195
H	1	-2.1435879975	1.8183378091	-0.8125254829
H	1	-2.2374922612	4.8325471355	-4.3430892074
H	1	0.8057451461	5.8589744825	-3.6379322859
H	1	0.9677891495	2.8801937388	-7.2005400940
H	1	-0.6527208048	0.6057951291	-5.4615594381
H	1	4.5407337588	2.6904746983	-4.2305371276
H	1	1.5417858594	-1.3731504917	-1.5695534931
H	1	6.7352385332	0.7115290775	-0.3385311826
H	1	5.1147304686	-1.5628695323	1.4004494733
H	1	4.5830598414	-0.2184579934	-7.8580948179
H	1	2.9483108018	-2.4806489733	-6.1165233476
H	1	7.4855580161	-3.5010745505	-6.1115401402
H	1	8.2261076156	-0.5010136025	-4.9875670275
H	1	5.2767725824	-4.5416502759	-2.1621583347
H	1	8.3200118794	-3.5152229289	-1.4570033030

Table S6: Atom types, atomic number, and coordinates in atomic units of the optimised structure of the silica $(SiO_2)_{10}$ cluster obtained from the calculations described in the main text.

Atom	Z	Coordinates / Bohr		
		X	Y	Z
O	8	-2.6835450731	4.9340443183	4.5792425950
O	8	2.6835242861	-4.9340480978	4.5792596025
SI	14	-2.9490383463	7.8885836570	3.5541834096
SI	14	2.9490402360	-7.8885836570	3.5541928583
O	8	-0.0000188973	-0.0000018897	4.8056620039
O	8	-0.4526082713	-3.6396028037	1.2895168887
O	8	0.4526007124	3.6395914654	1.2895093298
SI	14	-1.5058772862	2.3217343560	3.3759784695
SI	14	1.5058508300	-2.3217419150	3.3759954771
O	8	6.7948159245	5.7611962804	-5.4429361885
O	8	-6.7947970272	-5.7611981701	-5.4429664241
SI	14	4.6821646311	4.4500136833	-3.9342696667
SI	14	-4.6821438442	-4.4500136833	-3.9343017921
O	8	4.5252569029	1.4646755905	-3.1313969041
O	8	-4.5252398954	-1.4646737008	-3.1314309192
O	8	2.1030174702	5.7506648375	-2.8138643567
O	8	-2.1030023524	-5.7506553889	-2.8138794745
SI	14	2.9456840827	-0.7866324575	-1.7678273232
SI	14	-2.9456878621	0.7866419061	-1.7678518896

O	8	2.6964519016	-3.8574957690	-2.5029534091
O	8	-2.6964405633	3.8575014382	-2.5029817550
O	8	-3.4783770516	1.0027245139	1.3194142435
O	8	3.4783581544	-1.0027282933	1.3194406997
O	8	1.7316334013	-7.7620381562	0.6762365550
O	8	-1.7316258424	7.7620362665	0.6762289961
SI	14	0.4215317275	-5.5399811768	-1.0805037458
SI	14	-0.4215260583	5.5399773974	-1.0805094150
O	8	4.0288239962	-10.1852961512	4.9742633565
O	8	-4.0288221065	10.1852980410	4.9742501284
O	8	-0.0000000000	0.0000000000	-2.2327263723

Tables S7 through **S10** present the optimised geometries of the four optimised hydrocarbon-silica clusters considered in the paper.

Table S7: Atom types, atomic number, and coordinates in atomic units of the optimised structure of the benzene-silica $C_6H_6 \ldots (SiO_2)_{10}$ cluster obtained from the calculations described in the main text.

Atom	Z	Coordinates / Bohr		
		X	Y	Z
O	8	-3.6121129598	4.2698850021	4.5737850663
O	8	5.8523604415	-1.7365693656	4.2351178234
SI	14	-5.3275721964	6.7067641421	3.6014586847
SI	14	7.2929117900	-4.4482864737	3.3071300826
O	8	1.1383066843	1.2752778034	4.6094026217
O	8	2.3408243680	-2.1172867912	1.0334798043
O	8	-0.4059755031	4.6482251524	1.1333631611
SI	14	-1.3635015508	2.5476964513	3.2816471277
SI	14	3.5294128814	-0.0606356378	3.1044777574
O	8	3.8165510760	9.5166260591	-5.7969971392
O	8	-2.3689453804	-7.0966429592	-5.5229509662
SI	14	2.6635517722	7.3504785128	-4.2324079563
SI	14	-1.1184418847	-4.9483382057	-4.0098076836
O	8	4.0366342337	4.6881985262	-3.4622539095
O	8	-2.4346379249	-2.2792986693	-3.1488088394
O	8	-0.1797582949	7.2437656863	-3.0114919005
O	8	1.8011469718	-4.8194267680	-3.0085458177
SI	14	3.7829290713	1.9567545685	-2.0626680409
SI	14	-2.1101190605	0.4633834889	-1.8125968215
O	8	5.0372346956	-0.8294101846	-2.8711079363
O	8	-3.4426026490	3.2566290453	-2.5187552978
O	8	-2.5542575799	0.4292682656	1.2932906715
O	8	4.5131173032	2.0712946401	0.9879317388
O	8	6.2445522833	-4.7066800464	0.3103081250
O	8	-4.3493120751	7.1688909632	0.6665743860
SI	14	3.9769151131	-3.4142813256	-1.3430452670
SI	14	-2.2037663215	5.8397616384	-1.1735746404
O	8	7.6992539000	-6.7354690798	5.1070279455
O	8	-7.3138291670	8.2111126399	5.1032560524
O	8	0.8056506598	1.2562709394	-2.4255086587
C	6	11.2378810853	-2.8689668768	2.8641424063
C	6	10.9736558182	-0.7469349836	1.2723903021
C	6	11.6336331708	-0.8955260278	-1.2403178726
C	6	12.6518780042	-3.1341861397	-2.1799368768
C	6	13.0238856827	-5.2218590349	-0.6311873772
C	6	12.3701557643	-5.0835424309	1.8905329007
H	1	11.2139892796	-2.5915739990	4.8788000688
H	1	10.2255756607	0.9700700488	2.0326724203

H	1	11.3894012047	0.6942002902	-2.4609410209
H	1	13.1622533093	-3.2448069195	-4.1342291324
H	1	13.8229147427	-6.9172947349	-1.3822570813
H	1	12.6236077035	-6.6675372100	3.1179968571

Table S8: Atom types, atomic number, and coordinates in atomic units of the optimised structure of the cyclohexane-silica C_6H_{12}…$(SiO_2)_{10}$ cluster obtained from the calculations described in the main text.

Atom	Z	Coordinates / Bohr		
		X	Y	Z
O	8	-5.5092655706	2.7501220094	4.9624374513
O	8	-2.0474538570	-7.8521327324	3.7219854082
SI	14	-4.8522475289	5.7598507955	5.5641640003
SI	14	-1.2139240697	-10.2498794266	1.8763316099
O	8	-3.9510882280	-3.9510882280	4.4670571220
O	8	-1.8816474091	-4.6173394709	0.2812781544
O	8	-1.0165043958	1.5240186557	4.1571968620
SI	14	-3.9226308444	0.4024284874	3.6887640253
SI	14	-1.9474892420	-4.7592711206	3.4273922445
O	8	8.2819301213	3.2919933775	4.8156983386
O	8	-1.6199449263	-2.2593318245	-8.9058061486
SI	14	5.6705725809	2.3900781861	3.9014734722
SI	14	-1.1685819844	-2.2799940884	-6.0325325864
O	8	4.8979732287	-0.4485151248	2.9371576390
O	8	-1.5494600366	0.0711009403	-4.0565028128
O	8	3.1121840600	4.1024439413	3.5670448847
O	8	-0.1834394811	-4.6638872014	-4.3160472286
SI	14	2.7586238866	-2.3135480631	1.7704162478
SI	14	-1.3399706828	1.1096659973	-1.1745535185
O	8	3.0238752749	-4.7323330767	-0.2601491281
O	8	-0.5506434761	3.9768622008	-0.0832821140
O	8	-3.9040283817	0.5421208119	0.5510970103
O	8	0.9363516680	-3.6397294154	3.9577268354
O	8	-0.0212707557	-8.8376777473	-0.6456456707
O	8	-1.9897038308	6.1245131091	4.3620790639
SI	14	0.3673872985	-5.8750636095	-1.5498379818
SI	14	0.0993901383	4.2481323663	3.0046548718
O	8	-1.5275826789	-13.0950281970	2.3915899669
O	8	-6.5363070785	7.7310428706	6.8850730171
O	8	1.0416887740	-0.4674293922	-0.0077440971
C	6	2.8882477510	0.0308592254	12.3786652004
C	6	0.0746422868	-0.5432395297	12.7698687155
C	6	-1.4499565148	1.8721534258	13.2621744606
C	6	-1.0468080417	3.8267877217	11.1603210616
C	6	1.7654330403	4.4037078377	10.7602037090
C	6	3.2881647926	1.9844503926	10.2746896391
H	1	3.9058822029	-1.6899573844	11.9431457212
H	1	3.6824410021	0.7583734950	14.1241181678
H	1	-0.1655022020	-1.8521563454	14.3238773226
H	1	-0.6604082101	-1.4787370416	11.0953031492
H	1	-3.4399135689	1.4299915597	13.4411182836
H	1	-0.8678018578	2.6899550237	15.0505620025
H	1	-2.0712077127	5.5468087568	11.5864467129
H	1	-1.8226917378	3.0626637905	9.4120032695
H	1	2.0126847886	5.7295887235	9.2188827222
H	1	2.5012696632	5.3313686559	12.4331894643
H	1	5.2777306734	2.4324269455	10.0983838736
H	1	2.6710369768	1.1459638540	8.4969450339

Table S9: Atom types, atomic number, and coordinates in atomic units of the optimised structure of the naphthalene-silica $C_{10}H_8\ldots(SiO_2)_{10}$ cluster obtained from the calculations described in the main text.

Atom	Z	Coordinates / Bohr		
		X	Y	Z
O	8	-2.7852709128	4.5658312096	3.6545316390
O	8	6.0895890831	-2.2648214785	3.7387113729
SI	14	-4.3018837323	7.0488631273	2.4911123861
SI	14	7.2362596946	-5.1993335011	3.0590676419
O	8	1.6854107037	1.1792287006	3.8859040198
O	8	2.5005647954	-2.6099912685	0.6048086921
O	8	0.3577232397	4.3696058425	0.1491466236
SI	14	-0.7212800636	2.5452983890	2.4842451218
SI	14	3.9183902992	-0.4997663833	2.4715801782
O	8	4.8791326606	8.2451673920	-7.1639776756
O	8	-2.7826385245	-7.7082773416	-5.3791673850
SI	14	3.5451939831	6.3022002643	-5.4545107601
SI	14	-1.2998801460	-5.5601747887	-4.0909506278
O	8	4.6938998295	3.5972786108	-4.5017694986
O	8	-2.3515523424	-2.7125240211	-3.4673297135
O	8	0.7188706630	6.5517121248	-4.2154495553
O	8	1.6413082786	-5.6119003685	-3.1561485351
SI	14	4.2329503077	1.0277010223	-2.8603194906
SI	14	-1.7612737534	0.0993693513	-2.3926822285
O	8	5.1843498638	-1.9217039309	-3.4590735007
O	8	-2.8738026856	2.9263427372	-3.3248746096
O	8	-2.1417889783	0.3814789851	0.7089099173
O	8	5.0146468009	1.3505758251	0.1569360741
O	8	6.1862069934	-5.5630377236	0.0435808607
O	8	-3.3637217068	7.1668802947	-0.4903120842
SI	14	3.9713045166	-4.2541227977	-1.6613601610
SI	14	-1.3809720676	5.4933937621	-2.2336315510
O	8	7.1158274575	-7.4019886638	5.0164250330
O	8	-6.1026565383	8.8584401668	3.8875594197
O	8	1.1906823298	0.5955225786	-3.1195653297
C	6	11.1723397189	-4.4788149971	2.4639626928
C	6	11.8122330636	-4.2315538002	5.0433404003
C	6	12.4247518378	-1.8935904774	6.0806676867
C	6	12.5906716692	0.1746598141	4.5249262009
C	6	12.2148996566	0.0226729324	1.8778263832
C	6	11.5593537114	-2.3303704038	0.8087592590
C	6	11.3486095795	-2.5146338054	-1.8344061492
C	6	12.5734713833	2.1499091309	0.2979814424
C	6	12.3443043127	1.9256062151	-2.2582679088
C	6	11.7389494898	-0.4389568908	-3.3254679836
H	1	11.4142284247	-6.3266552083	1.6349758068
H	1	11.6828567533	-5.8676804501	6.2229319283
H	1	12.7729716455	-1.7251724282	8.0610192636
H	1	13.0694885503	1.9751340435	5.3198148712
H	1	10.8551737783	-4.2871948922	-2.6672707529
H	1	13.0420553981	3.9309343003	1.1369933248
H	1	12.6249267322	3.5259168883	-3.4599371054
H	1	11.5761363679	-0.6074599777	-5.3338423072

Table S10: Atom types, atomic number, and coordinates in atomic units of the optimised structure of the trans-decalin-silica $C_{10}H_{18}\ldots(SiO_2)_{10}$ cluster obtained from the calculations described in the main text.

Atom	Z	Coordinates / Bohr		
		X	Y	Z
O	8	1.7199435564	8.5550446594	2.0971347532
O	8	6.0339971240	-1.5610818515	4.3276236900
SI	14	0.5878484014	10.6464970066	0.0491234271
SI	14	5.6412610411	-4.5082248026	5.3320357291
O	8	4.0535416122	3.5854205802	3.3119545531
O	8	1.4208755215	-0.7472619062	3.7129846433
O	8	1.5700523807	4.9174581933	-0.9154172835
SI	14	1.8319098211	5.4497599890	2.1785025748
SI	14	4.1976332187	0.5004939278	2.9029082452
O	8	0.8823584171	1.4342358843	-9.7137792817
O	8	-7.6316527322	-3.0018278418	5.3246809156
SI	14	0.6209223856	1.7786592325	-6.8374878270
SI	14	-5.1743853386	-2.0722054880	4.0738051439
O	8	1.3067096157	-0.2807036777	-4.6316503658
O	8	-4.6866073769	0.6657542449	2.7137636814
O	8	-0.4259574657	4.2847968299	-5.3465979576
O	8	-2.5322290441	-3.6485336487	3.7347750736
SI	14	1.3642026392	-0.9693236070	-1.6397568135
SI	14	-2.7762115664	2.5110849000	1.1767134753
O	8	0.8629811668	-3.7263941389	-0.1639091630
O	8	-3.3560513090	4.6380962211	-1.1002022495
O	8	-0.9188169006	4.2150300362	3.0495868867
O	8	3.9861558732	-0.0263900234	-0.1864120201
O	8	2.7512350580	-5.2156512850	4.3773234834
O	8	-0.6592290211	8.8583267832	-2.1925073341
SI	14	0.4400793880	-3.6606467923	2.9745534266
SI	14	-0.8830519465	5.7876826799	-2.7093077075
O	8	7.5378883082	-6.1692315849	6.7853956404
O	8	0.7197720623	13.5479841779	0.1995871896
O	8	-1.0464867883	0.5791481029	-0.4943995615
C	6	-3.3254698733	-1.0094727254	15.7810110166
C	6	-5.5438475562	-0.8281648552	13.9299374842
C	6	-4.7609945507	0.4690488874	11.4635143682
C	6	-2.4551981437	-0.8302378846	10.2832954512
C	6	-0.2234468700	-0.9945212134	12.1324773679
C	6	-1.0228652135	-2.3215529424	14.6032940968
C	6	1.2021718638	-2.4873915156	16.4559115353
C	6	2.0877069103	-2.3043167516	10.9627464301
C	6	4.3075377924	-2.4815636006	12.8172649330
C	6	3.5090359661	-3.7802173145	15.2781076881
H	1	-2.7843354984	0.8777134706	16.3844420985
H	1	-3.9012467051	-2.0199810206	17.4669791894
H	1	-7.0978731708	0.1878104173	14.7907397970
H	1	-6.2374431341	-2.7116830931	13.5082281226
H	1	-6.3296806596	0.4631227067	10.1495519842
H	1	-4.3051019356	2.4340615585	11.8399685720
H	1	-1.8570828610	0.1868107522	8.5991244078
H	1	-2.9770081806	-2.7348095597	9.7108596550
H	1	0.3287953143	0.9272828730	12.6165136721
H	1	-1.5772371189	-4.2425860205	14.1134298777
H	1	1.7163417386	-0.5919698937	17.0567782590
H	1	0.6175303274	-3.4963086615	18.1396989644
H	1	2.6548155689	-1.2673655207	9.2794635579
H	1	1.5485945421	-4.1967393783	10.3664887481
H	1	5.8685648419	-3.4951937232	11.9667691857

H	1	4.9852955768	-0.5906584239	13.2362096255
H	1	3.0650202788	-5.7455096650	14.8913053449
H	1	5.0687364278	-3.7836112623	16.6027337959

Total Energies and Zero Point Energies

Table S11 below lists the total energies, corrected for BSSE using the CP method, and the harmonic zero-point energies used in calculating the binding energies of the various clusters discussed in the main paper.

Table S11: Summary of the computed energetics of the monomers and clusters considered in this work. Monomer energies are taken from the optimised cluster geometry with the relevant atoms ghosted as per the CP method.

Species	Total Energy / Hartree	Zero Point Energy / Hartree
Monomers		
C_6H_6	-230.6578167684	0.107805
C_6H_{12}	-234.1513845647	0.201188
$C_{10}H_8$	-383.2722274857	0.142585
trans-$C_{10}H_{18}$	-389.0937757093	0.281583
$(SiO_2)_{10}$	-4387.5073416721 (C_6H_6)<br>-4387.5174044652 (C_6H_{12})<br>-4387.5018172354 ($C_{10}H_8$)<br>-4387.5211627806 ($C_{10}H_{18}$)	0.113771
Clusters		
C_6H_6 - $(SiO_2)_{10}$	-4618.1943021628	0.223211
C_6H_{12} - $(SiO_2)_{10}$	-4621.6731391579	0.295939
$C_{10}H_8$ - $(SiO_2)_{10}$	-4770.8215399631	0.273656
trans-$C_{10}H_{18}$ - $(SiO_2)_{10}$	-4776.6159791857	0.395891

Results and Discussion

Cyclohexane

Figure S2 presents the results of the AA for the exposures displayed in the main text **Figure 2** but not reported in **Figure 4**. The activation energy values derived from the lines of best fit displayed in **Figure S2** are listed in **Table 1** in the main text.

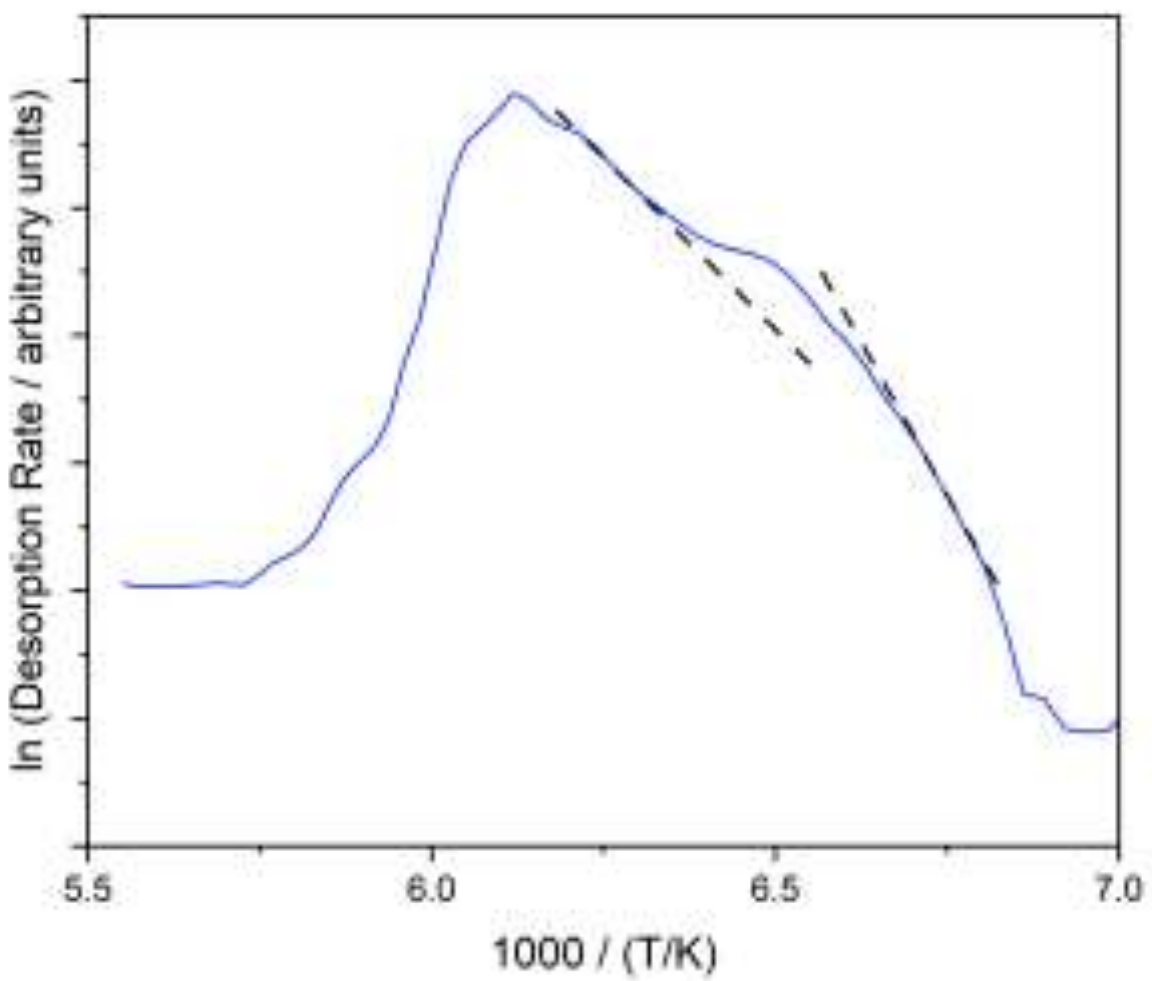
ln (Desorption Rate / arbitrary units)
5.5
6.0
6.5
7.0
1000 / (T/K)

(a)

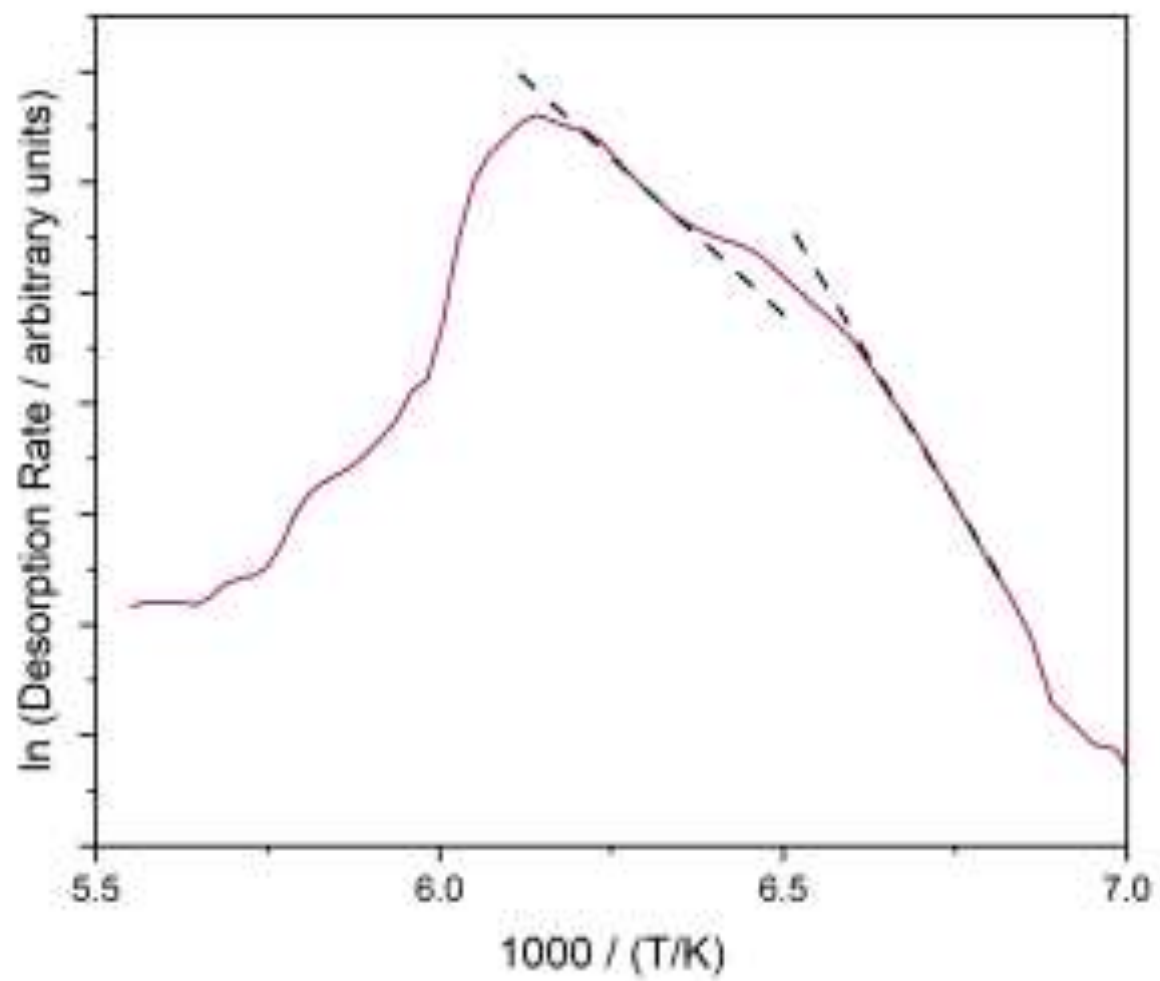
ln (Desorption Rate / arbitrary units)
5.5
6.0
6.5
7.0
1000 / (T/K)

(b)

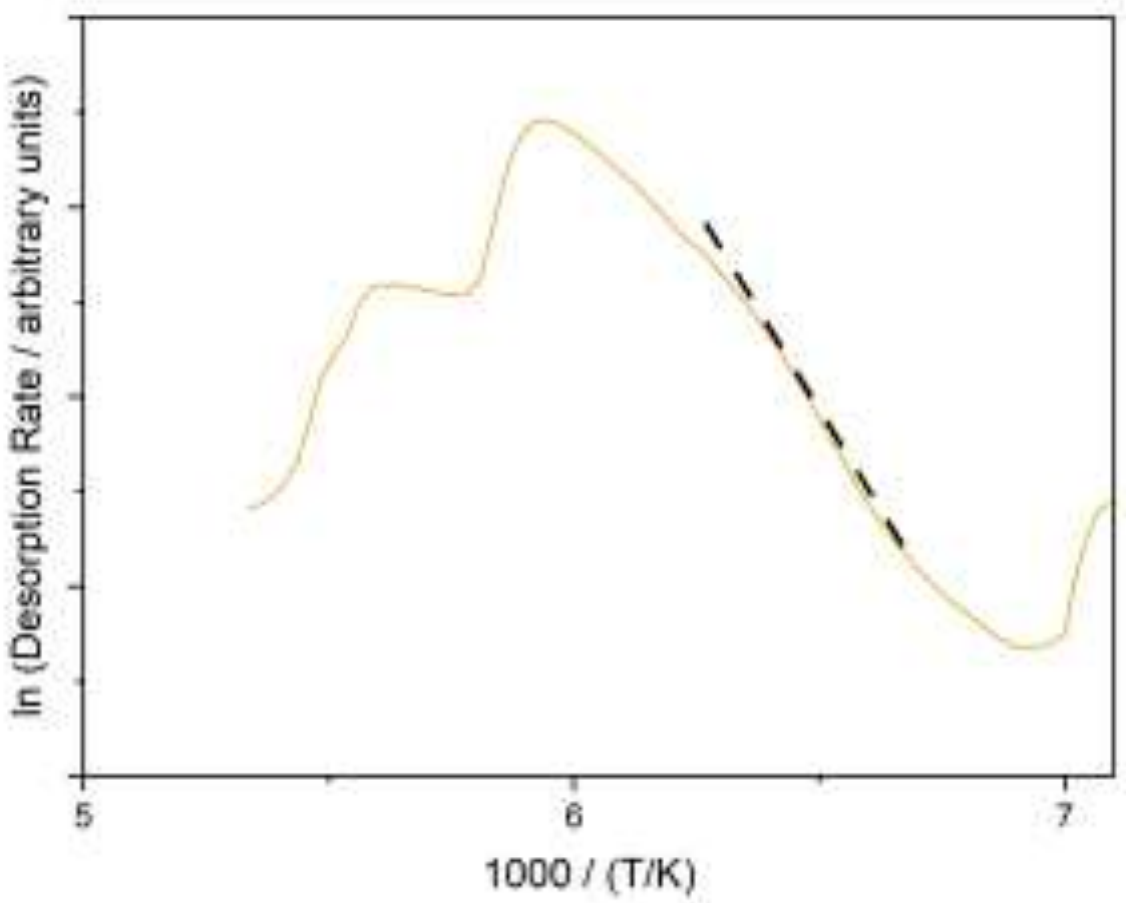
ln (Desorption Rate / arbitrary units)
5
6
7
1000 / (T/K)

(c)

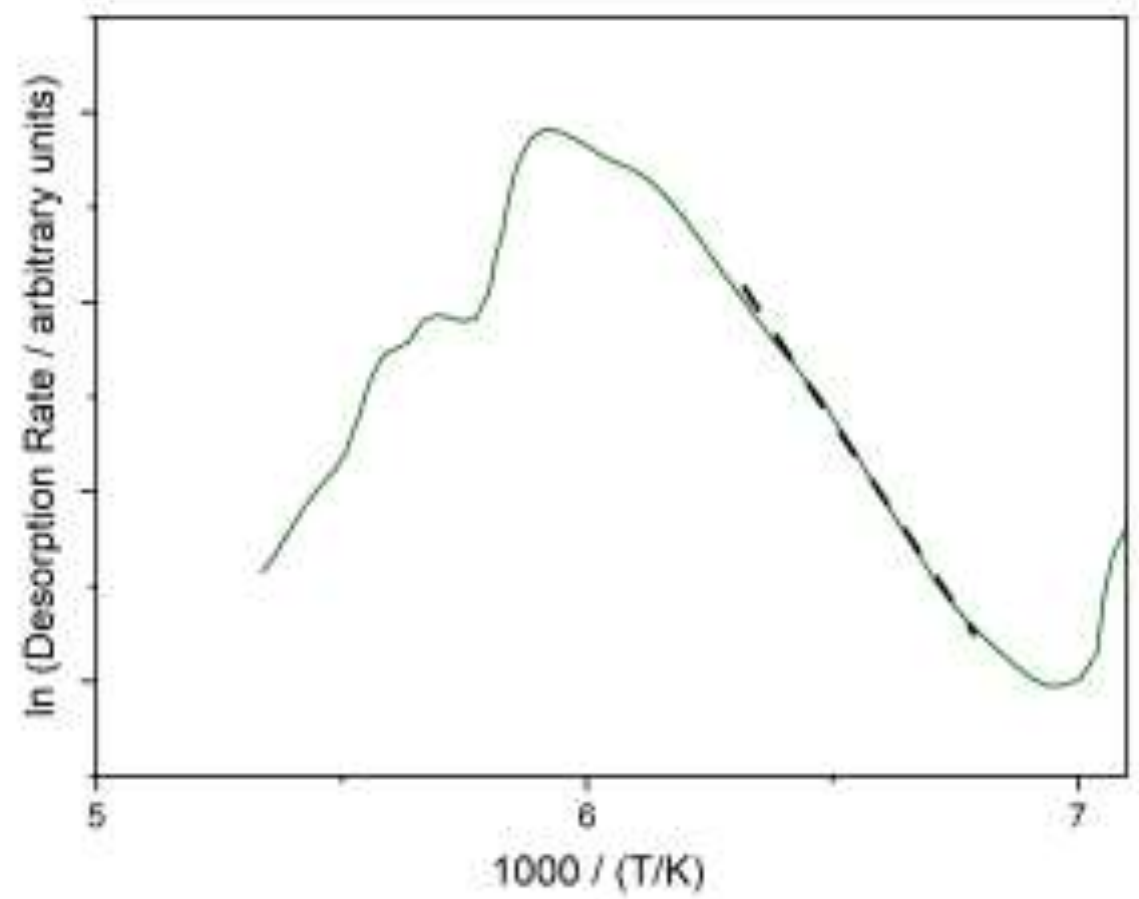
ln (Desorption Rate / arbitrary units)
5
6
7
1000 / (T/K)

(d)

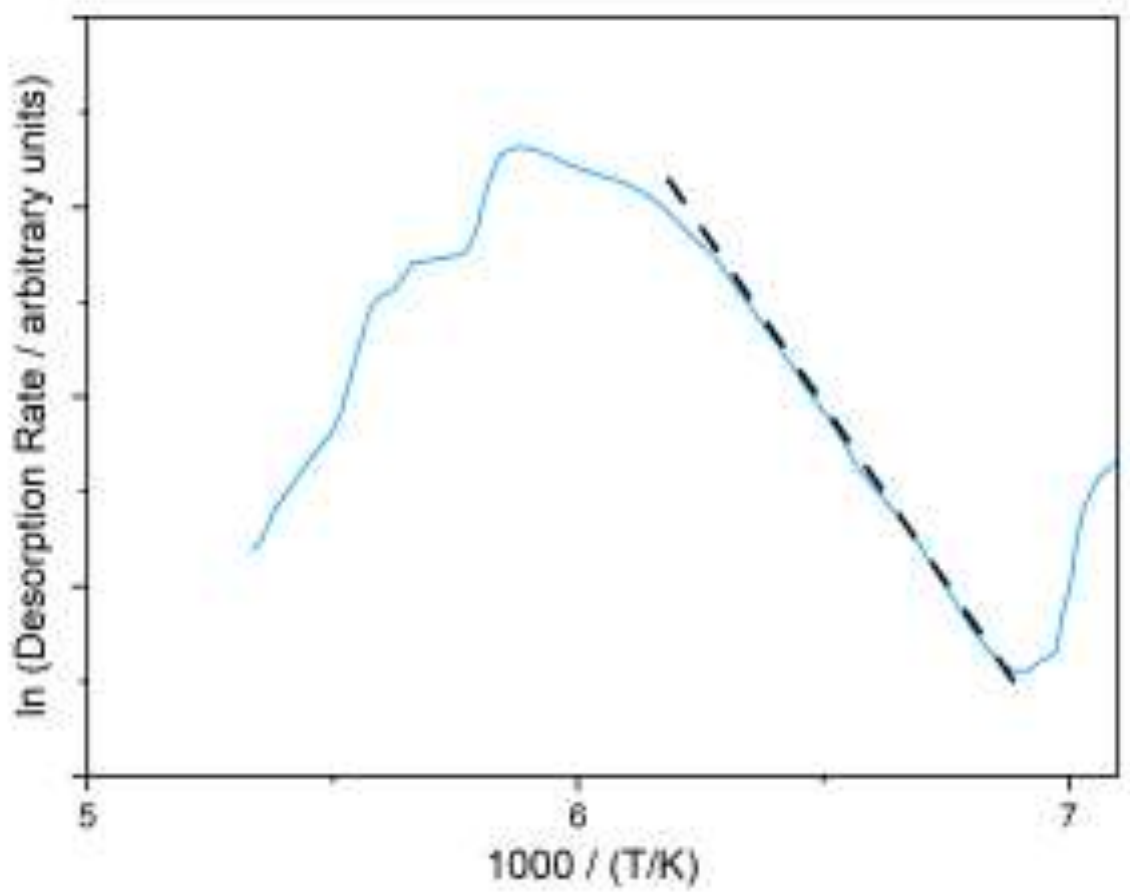

(e)

Figure S2: Arrhenius Analysis of the TPD of (a) 0.6 L, (b) 1.2 L, (c) 15 L, (d) 35 L, and (e) 125 L of C_6H_{12} deposited on an $aSiO_2$ surface at 115 L. The broken lines are the result of linear regression fits to the leading-edge data.

trans-Decalin

Figure S3 presents the Arrhenius semi-log plots of the required for the AA analysis of the thermal desorption of multilayer quantities of *trans*-$C_{10}H_{18}$. The corresponding E_{des} derived from the linear portions indicated by the straight lines are listed in **Table 3** in the main text.

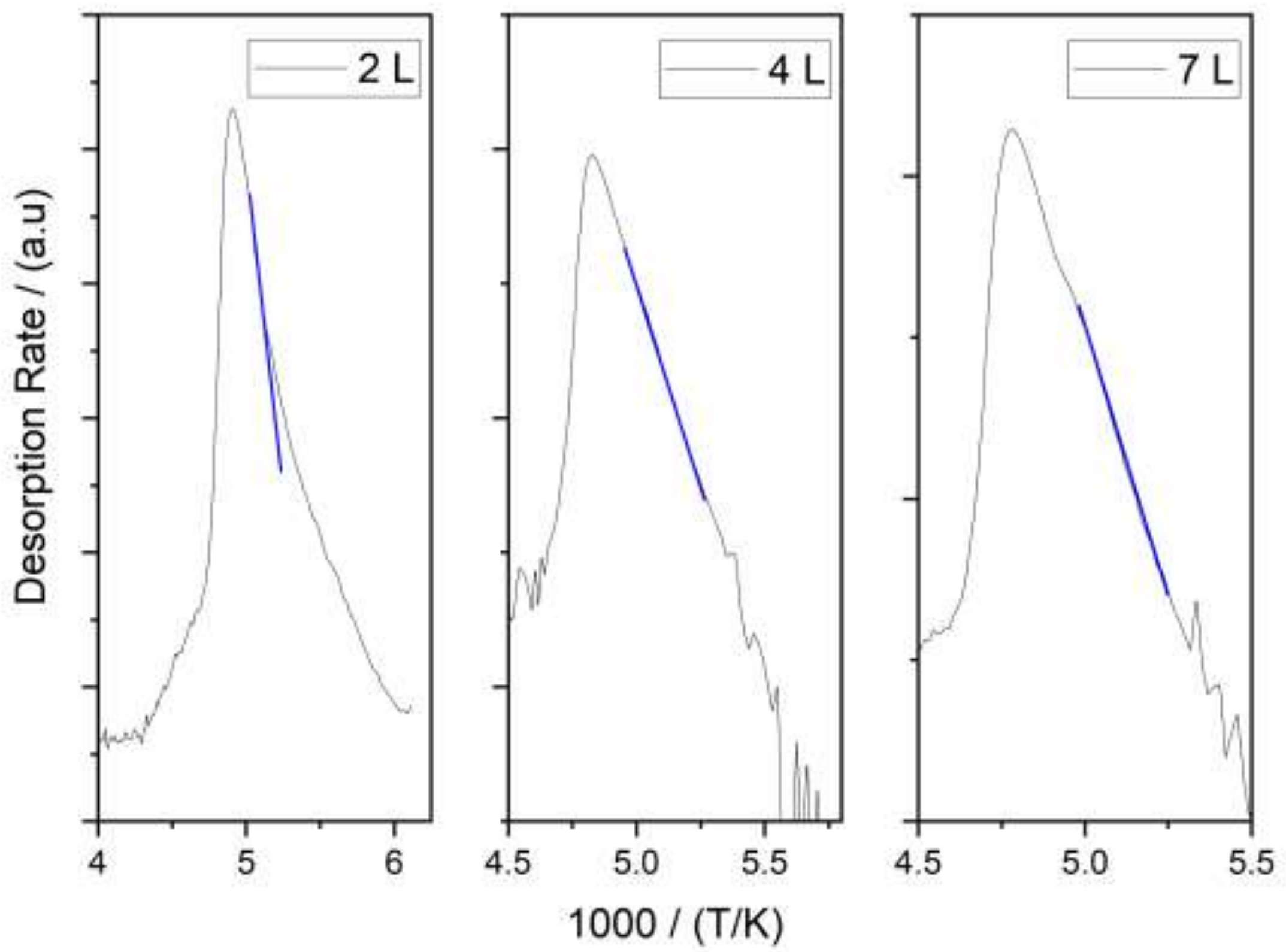

Figure S3: Arrhenius plots for the TPD of thin multilayer exposures (2, 4, and 7 L) of *trans*-$C_{10}H_{18}$ from an aSiO_2 substrate. The solid blue line represents the best linear fit to the leading edge of the TPD.

CKS simulations assuming a simple two step model as with multilayer C_6H_{12} were then used to arrive at an optimised ν_{des} using the AA derived E_{des} value, and assuming the experimental heating ramp data. **Figure S4** illustrates the results of these simulations. The optimised ν_{des} are reported in **Table 3**.

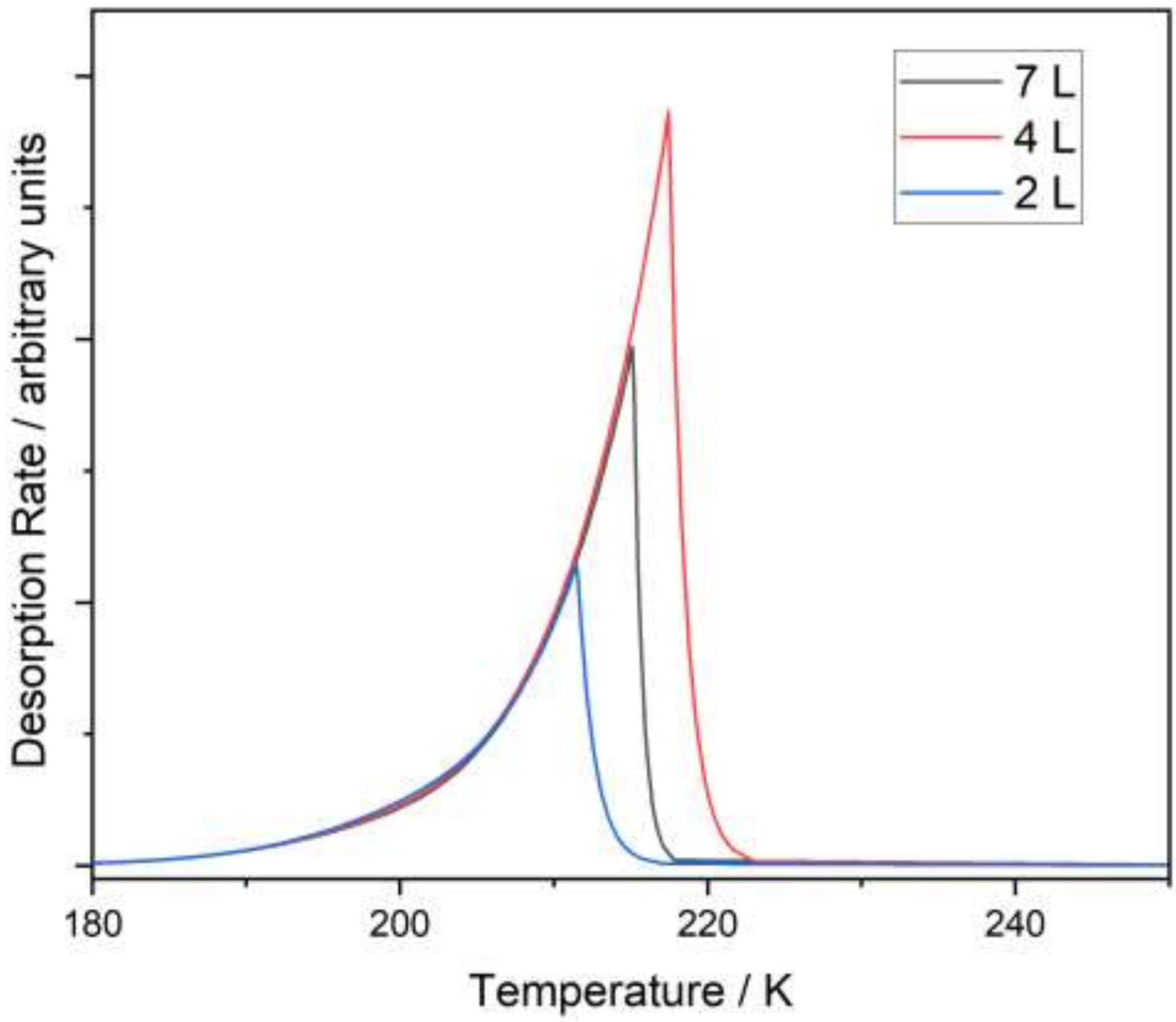

Figure S4: CKS simulations of the TPD of *trans*-$C_{10}H_{18}$ thin multilayers deposited on an $aSiO_2$ substrate (2, 4, and 7 L exposures) at 115 K.

References

[1] Bromley, S. T., and M. A. Zwijnenburg, *Computational Modeling of Inorganic Nanomaterials,* (CRC Press, Boca Raton, USA, 2016).

[2] Gordon, M. S., and M. W. Schmidt, in *Advances in Electronic Structure Theory: GAMESS a Decade Later*, ed. C. E. Dykstra, G. Frenking, K. S. Kim and G. E. Scuseria, (Elsevier, Amsterdam, Netherlands, 2005).

[3] Barca, G. M. J., C. Bertoni, L. Carrington, D. Dipayan, N. De Silva, J. E. Deustua, D. G. Fedorov, J. R. Gour, A. O. Gunina, E. Guidez, T. Harville, S. Irle, J. Ivanic, K. Kowalski, S. S. Leang, H. Li, W. Li, J. J. Lutz, I. Magoulas, J. Mato, V. Mironov, H. Nakata, B. Q. Pham, P. Piecuch, D. Poole, S. R. Pruitt, A. P. Rendell, L. B. Roskop, K. Ruedenberg, T. Sattasathuchana, M. W. Schmidt, J. Shen, L. Slipchenko, M. Sosonkina, V. Sundriyal, A. Tiwari, J. L. Galvez Vallejo, B. Westheimer, M. Wloch, P. Xu, F. Zahariev, and M. S. Gordon, "*Recent developments in the general atomic and molecular electronic structure system*", *J. Chem Phys.*, 2020, **152**, 154102, DOI:10.1063/5.0005188.

[4] Allouche, A. R., “*Gabedit—A graphical user interface for computational chemistry softwares*”, *J. Comput. Chem.,* 2011, **32**, 174-182, DOI:10.1002/jcc.21600

[5] Jansen, H. B., and P. Ros, “*Non-empirical molecular orbital calculations on the protonation of carbon monoxide*”, *Chem. Phys. Lett.*, 1969, **3**, 140-143, DOI:10.1016/0009-2614(69)80118-1.

[6] Liu, B., and A. D. Mclean, “*Accurate calculation of the attractive interaction of two ground state helium atoms*”, *J. Chem. Phys.*, 1973, **59**, 4557-4561, DOI:10.1063/1.1680654.

[7] Van Duijneveldt, F. B., J. G. C. M. van Duijneveldt-van de Rijdt, and J. H. van Lenthe, "*State of the art in counterpoise theory*", *Chem. Rev.*, 1994, **94**, 1873–1885, DOI:10.1021/cr00031a007
